# Coordination-Driven Scrambling in Tricopper(I) Clusters: Thermodynamic Insights into Dynamic Assembly

**DOI:** 10.1021/acs.inorgchem.6c02605

**Published:** 2026-07-25

**Authors:** Venkata Sai Sashankh Penki, Najeeb Ullah, Sri Sudewi, Rahimeh Eshaghi Malekshah, Hsing Yin Chen, Ya-Fan Lin, Sodio C. N. Hsu

**Affiliations:** † Department of Medicinal and Applied Chemistry, 38023 Kaohsiung Medical University, Kaohsiung 80708, Taiwan; ‡ Department of Chemistry Education, Faculty of Mathematics and Natural Sciences, 175463 Yogyakarta State University, Yogyakarta 55281, Indonesia; § Department of Pharmacy, Faculty of Mathematics and Natural Science, Universitas Sam Ratulangi, Manado 95115, Indonesia; ∥ Department of Chemistry, 63373 National Dong Hwa University, Hualien, 974301, Taiwan; ⊥ Department of Medical Research, Kaohsiung Medical University Hospital, Kaohsiung 80708, Taiwan

## Abstract

Controlling product distributions in multicomponent self-assembly remains a central challenge in coordination chemistry, particularly when exchange occurs at the level of pre-organized metal–ligand fragments. *β-*Thioketiminato tricopper­(I) clusters, [**L**Cu]_3_, define a rare class of metal-cluster-based dynamic combinatorial systems that redistributes Cu–ligand coordination units rather than free ligand exchange. Mixing homoleptic precursors [**L**_**A**_Cu]_3_ (**A3**) and [**L**_**B**_Cu]_3_ (**B3**) generates a dynamic library of heteroleptic species (**A2B** and **AB2b**), those distributions follow binomial statistics across varying initial compositions. Variable-temperature NMR (VT-NMR) establishes rapid, reversible exchange and reveals temperature-dependent equilibration. Non-linear Van't Hoff analysis demonstrates that increasing temperature enhances the entropic stabilization of heteroleptic assemblies. In contrast, lower temperatures promote enthalpy-driven homoleptic stabilization with a thermodynamic regime crossover near 275 K. Complementary DFT calculations suggest that low-temperature scrambling proceeds through enthalpy-biased redistribution involving dicopper fragments and monomeric intermediates. In comparison, higher temperatures enable more entropy-driven pathways through solvation-assisted dissociation. These results show that simplified multinuclear clusters provide a model for studying thermodynamic control of entropy-driven self-assembly and tunable self-sorting in metal-cluster systems.

## Graphical abstract

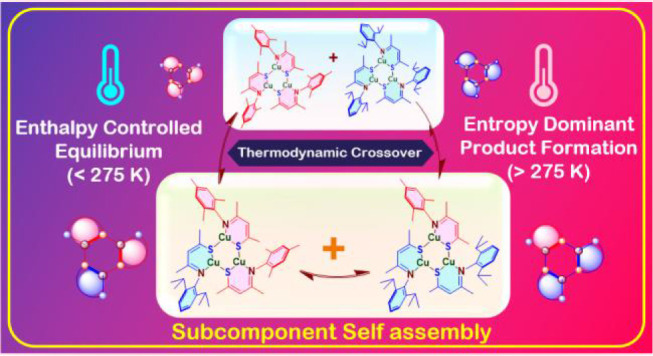

## Introduction

Coordination-driven self-assembly has emerged as a powerful strategy for constructing discrete supramolecular architectures with well-defined geometries and functional properties. Inspired by the structural sophistication of biological systems, this approach has led to breakthroughs in catalysis, molecular recognition, sensing, and materials design.^1,2^ Pioneering works by Lehn,^3^ Stang,^4^ Fujita,^5^ Mirkin,^6^ Raymond,^7^ and others^8–10^ have demonstrated that metal-ligand coordination, with its directional bonding and predictable geometry, can reliably generate complex and highly ordered supramolecular structures. Traditionally, this process leads to single, thermodynamically favored products, termed absolute self-organization.^11^ Hahn and coworkers introduced trans-metalation using strategic templates, synthesizing SN chelating Schiff-based dinickel and dizinc complexes, followed by trans-metalation with palladium acetate to give stable dipalladium helicase complexes.^12^ More recently, Nitschke’s subcomponent self-assembly concept^11^ has expanded the field, leveraging *in situ* formation of dynamic covalent and coordination bonds around metal centers to build complex architectures from simpler precursors. Synthetically, coordination-driven self-assembly outcomes generally fall into three organizational regimes: statistical distributions, amplified self-organization, or absolute self-organization.^13,14^ While Severin and coworkers have demonstrated Dynamic Combinatorial Libraries (DCLs),^15,16^ based on molar-dependent competitive mechanisms in trimeric metal clusters via DCL strategies,^17,18^ achieving such control remains challenging when directing forces are weak. Despite the remarkable structural diversity achieved in coordination-driven self-assembly, understanding the thermodynamic origins governing these organizational regimes remains challenging. In the absence of geometric, electronic, or templating biases, self-assembly frequently produces statistically distributed “scrambled” ensembles rather than a single well-defined heteroleptic structure.^6,11,19–22^ Dynamic ligand exchange processes have also been explored in metal–organic cages as probes of kinetic stability and adaptive behavior, although the equilibrated product distributions in weakly biased systems frequently approach near-statistical ensembles exchanging fragments *via* reversible covalent or coordination bonds.^17,23,24^ At equilibrium, the subcomponents in the dynamic combinatorial libraries favor constituents with strong binding interactions or greater geometric complementarity, leading to selective product amplification.^25,26^ Severin and coworkers demonstrated that subtle concentration-dependent competitive effects can influence product selection in trimeric metal clusters,^15,17^ whereas Clever and coworkers highlighted that ligands of similar dimensions often lead to thermodynamic “dead-ends” characterized by near-statistical product mixtures.^14^ Such systems are often interpreted as arising from configurational degeneracy competing effectively against only subtle energetic differentiation among competing assemblies. However, while statistical distributions are commonly observed in coordination-driven self-assembly, the thermodynamic origins governing the transition between statistical and non-statistical ensemble behavior remain considerably less explored. Rather than representing trivial random outcomes, near-statistical distributions may instead reflect shallow free-energy landscapes in which configurational entropy competes against only subtle energetic differentiation among accessible assemblies. However, experimentally resolving these subtle thermodynamic contributions remains highly challenging in most supramolecular systems. A major challenge arises from the structural and compositional complexity of higher-order supramolecular systems, where overlapping equilibria and signal congestion often obscure direct thermodynamic interpretation. In contrast, structurally simplified multinuclear clusters provide unusually tractable platforms for experimentally interrogating ensemble-level thermodynamics. Unlike high-nuclearity cages that may involve multiple topologies or oligomeric intermediates, small multinuclear systems with fixed nuclearity and conserved coordination frameworks permit direct observation of population redistribution among closely related ensemble states. Such systems therefore provide a unique opportunity to examine how configurational entropy, subtle enthalpic differentiation, solvation effects, and conformational flexibility collectively shape the free-energy landscape of self-assembly. Herein, we employ the well-defined *β-*thioketiminato tricopper­(I) thiolate assemblies, [**L_A_** Cu]_3_ (**A3**) and [**L_B_** Cu]_3_ (**B3**), as a precision model for probing temperature-dependent ligand scrambling within a shallow ensemble free-energy landscape (see Scheme 1). By combining variable-temperature NMR spectroscopy with Density Functional Theory (DFT) analysis, these results support a temperature-dependent transition between distinct redistribution regimes and demonstrate that simplified multinuclear clusters can serve as effective model systems for probing the thermodynamic factors governing statistical self-assembly in dynamic coordination systems.

**Scheme 1:**
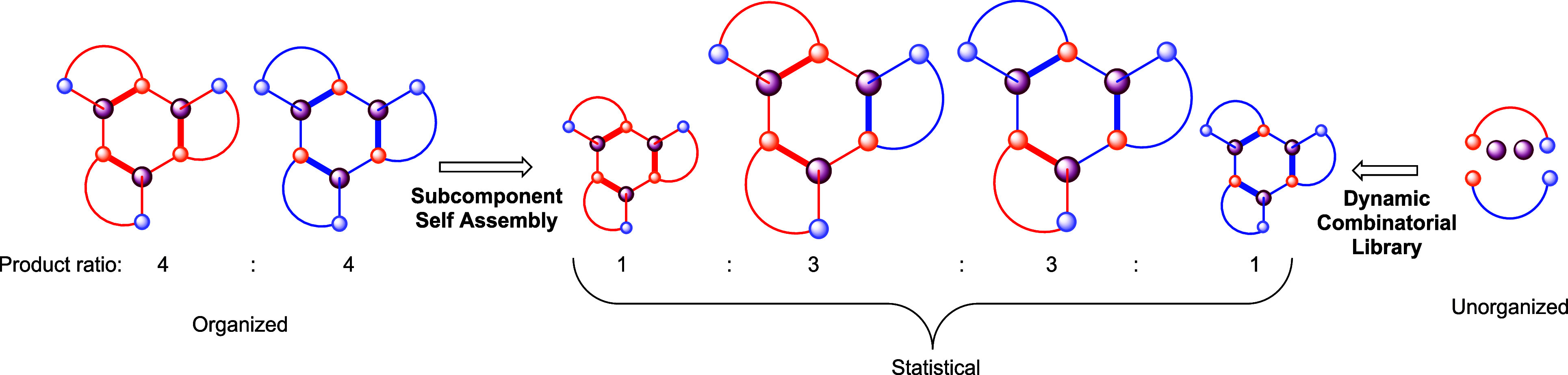
Representation of Subcomponent Self-Assembly and the Formation of Dynamic Combinatorial Libraries in the *β-*Thioketiminato tricopper­(I) Clusters

## Results and Discussion

### Synthesis of *β-*Thioketiminato Tricopper­(I) Clusters

*N*-Aryl-substituted *β-*thioketiminato ligands were synthesized according to previously reported procedures, wherein *β-*ketiminato ligands were treated with 0.5 equivalents of Lawesson’s reagent, followed by purification *via* column chromatography.^27–30^ These ligands were subsequently reacted with CuO^*t*^Bu in a 1:1 stoichiometric ratio to afford tricopper clusters, as illustrated in Scheme 2 and detailed in the Supporting Information. The selection of specific ligand sets and their corresponding complexes was informed by their distinct physicochemical characteristics.^31^ Complexes [**L**_**A**_Cu]_3_ and [**L**_**B**_Cu]_3_, designated as **A3** and **B3**, respectively, display similar electron-donating properties, as evidenced by FTIR stretching frequencies using CO and isocyanide ligands as spectroscopic probes.^27,28^

**Scheme 2:**
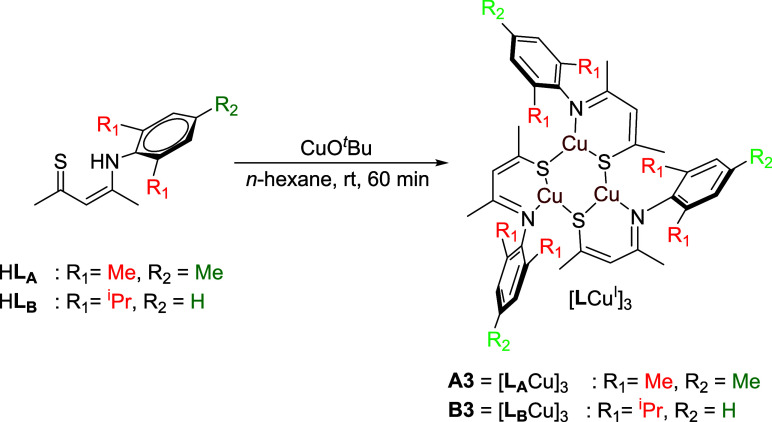
Synthesis of Tricopper Clusters from the *β-*Thioketiminate Ligands

In *β-*thioketiminato-supported tricopper­(I) clusters, each Cu­(I) center forms covalent bonds with adjacent ligand donors and is further bridged to form a cyclic hexameric chair-like structure. Previous studies using CO and CNR as spectroscopic probes suggested that the bridging Cu–S bonds are more labile than the chelating Cu–S bonds, allowing for selective formation of **L**CuCO or **L**CuCNR adducts.^27,28^ To further understand the dynamic nature of these Cu–S interactions, we investigated subcomponent exchange (scrambling) in solutions of [**L**_**A**_Cu]_3_ (**A3**) and [**L**_**B**_Cu]_3_ (**B3**) under mild conditions. Combining individual solutions of these complexes in CH_2_Cl_2_, CHCl_3_, or THF spontaneously generated a series of new scrambling products, reflecting dynamic equilibrium behavior. Both [**L**Cu]_3_ clusters (**A3** and **B3**) were soluble in these solvents and capable of self-assembly into thermodynamically favored tricopper clusters. In this study, **L**_**A**_H and **L**_**B**_H designate the free ligands, while **L**_**A**_Cu (**A**) and **L**_**B**_Cu (**B**) correspond to the pre-organized copper­(I)–ligand coordination units that serve as the fundamental building blocks for cluster formation and dynamic redistribution.

### NMR Investigation of Dynamic Subcomponent Exchange and Scrambling Behavior in Tricopper­(I) Clusters

Upon mixing [**L**_**A**_Cu]_3_ (**A3**) and [**L**_**B**_Cu]_3_ (**B3**) in equimolar amounts, the reaction afforded a mixture of subcomponent-exchanged scrambling products, collectively referred to as **SP1**. This mixture is proposed to consist of four complexes: [**L**_**A**_Cu]_3_ (**A3**), [(**L**_**A**_Cu)_2_(**L**_**B**_Cu)] (**A2B**), [(**L**_**A**_Cu)­(**L**_**B**_Cu)_2_] (**AB2**), and [**L**_**B**_Cu]_3_ (**B3**), in an approximate statistical ratio of 1:3:3:1. Notably, the same product distribution was also obtained *via* an alternate synthetic strategy (Path B of Scheme 3), in which equimolar amounts of ligands **L**_**A**_H and **L**_**B**_H were treated with two equivalents of CuO^*t*^Bu under dynamic combinatorial conditions.

**Scheme 3:**
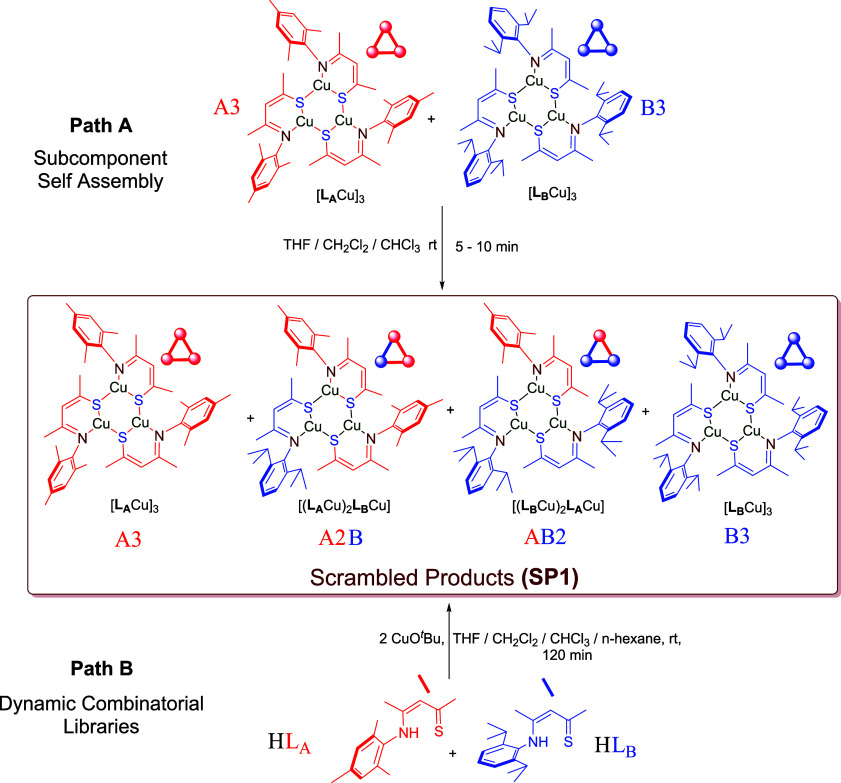
Schematic Representation of the Formation of Scrambled Products (**SP1**) *via* Two Pathways: Path A: Subcomponent Exchange Resulting from the Direct Mixing of Two *β*-Thioketiminato copper (I) Clusters, [**L**_**A**_Cu]_3_ and [**L**_**B**_Cu]_3_. Path B: One-Pot Synthesis of **SP1** from *β*-Thioketiminato Ligands H**L**_**A**_ and H**L**_**B**_ in the Presence of CuO^*t*^Bu, Leading to *In Situ* Formation and Scrambling of Copper­(I) Clusters

This pathway also led to the *in situ* formation of **SP1**, confirming that the same thermodynamically favored mixture can arise regardless of the synthetic route. Due to rapid equilibration, it was not feasible to isolate the individual components from **SP1**. Instead, the scrambling behavior was monitored using ^1^H NMR spectroscopy in CDCl_3_. Each peak was assigned based on reactions monitored by stoichiometric ratios and their relative intensities observed in the ^1^H NMR spectra. Initially, aryl proton signals of **A3**, **A2B**, **AB2**, and **B3** merged into two sets, corresponding to **A3** and **B3** aryl components at 6.80 and 7.09 ppm, respectively.^28^ In addition, the −CH_3_ groups (*ortho*-ArCH_3_ and *para*-ArCH_3_) of **A3** appeared well-defined but broadened, overlapping with the backbone methyl group protons, which complicates analysis. This may result from the ArCH_3_ groups being farther away from the Cu–S core, reducing their role in subcomponent self-assembly as observed in the ^1^H NMR spectra. Furthermore, the backbone −CH_3_ protons, such as those in NCCH_3_ and SCCH_3_ groups, merged with the isopropyl group protons at 1.75 to 1.00 ppm, further complicating peak assignments in the **SP1** mixtures of the ^1^H NMR spectra.

The methine (−C*H*) protons on the *β-*thioketiminato backbone served as a sensitive spectroscopic probe for identifying each component. For the pure complexes, **A3** and **B3**, sharp singlet resonances were observed at 5.81 and 5.94 ppm, respectively (Figure 1a).^28^ Upon mixing, the ^1^H NMR spectrum of the **SP1** mixture displayed seven distinct singlets in the range of 5.78 to 6.05 ppm, corresponding to eight chemically distinct −C*H* environments. This distribution is consistent with a statistical analysis of the four proposed species and reflects the formation of a dynamic equilibrium among the scrambled products. These findings provide compelling evidence for spontaneous subcomponent exchange between **A3** and **B3** and highlight the kinetic lability of the bridging Cu–S bonds that facilitate rapid ligand reorganization. The ability of the system to access all possible combinatorial assemblies highlights the dynamic, reversible nature of *β*-thioketiminato-supported tricopper­(I) clusters under mild conditions.

**Figure 1:**
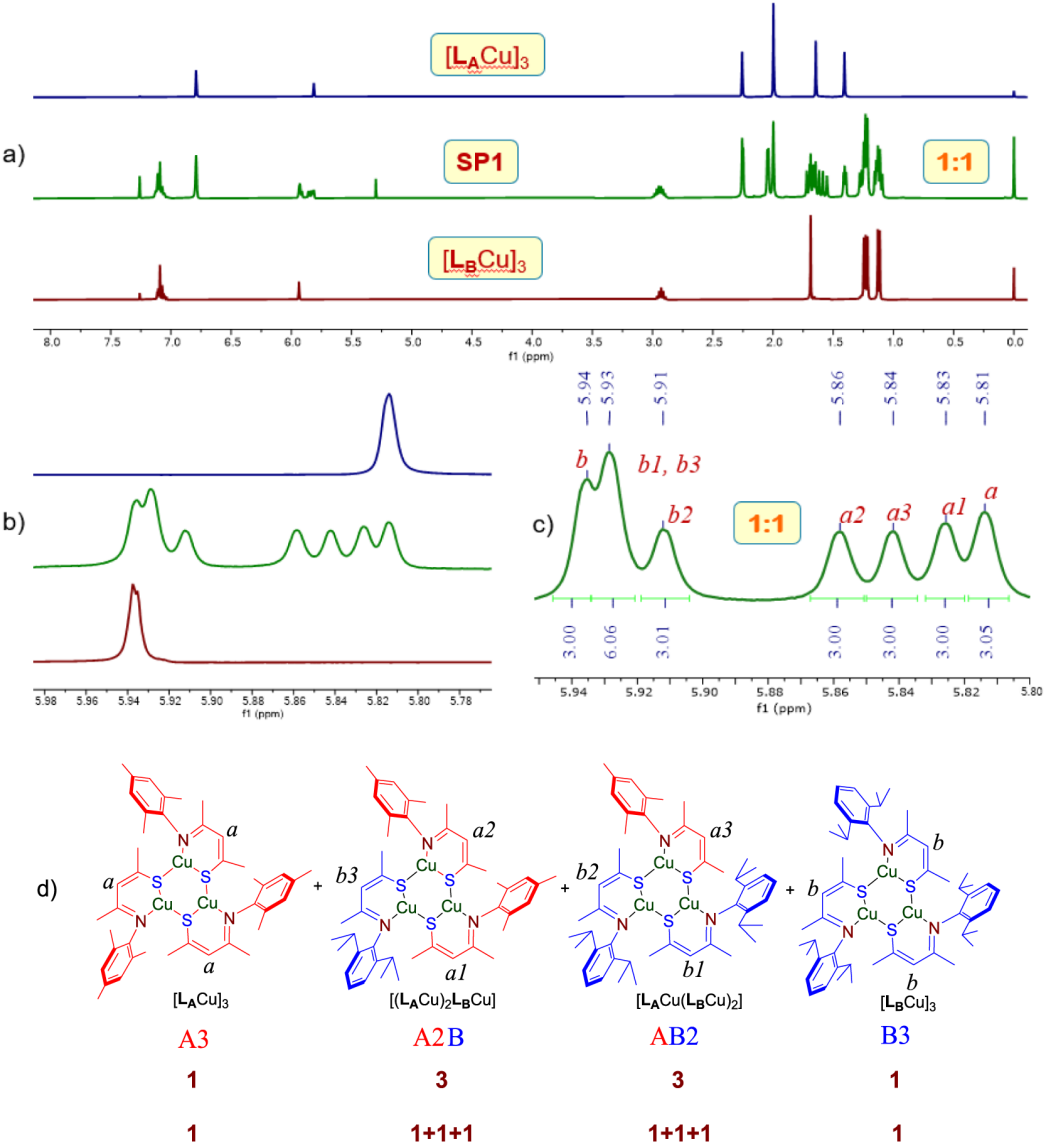
(a) Comparison of ^1^H NMR of the subcomponent-exchanged product mixture **SP1** (1:1 ratio, green trace) with the parent trimers, [**L_A_** Cu]_3_ (**A3**, blue trace) and [**L_B_** Cu]_3_ (**B3**, red trace). (b) Expanded view of the methine (−C*H*) backbone region (5.80 to 6.00 ppm), highlighting differences between **A3**, **B3**, and **SP1**. (c) Assignment of individual −C*H* proton resonances in the **SP1** mixture corresponding to the four major species: **A3**, **A2B**, **AB2**, and **B3**. (d) Product distribution of **SP1** based on integration of the methine (−C*H*) signals.

### Statistical Self-Assembly and Binomial Modeling of Scrambled Tricopper­(I) Clusters

To gain quantitative insights into the self-assembly behavior of the *β*-thioketiminate tricopper­(I) clusters, the ^1^H NMR spectra of **SP1** mixtures were analyzed, with particular focus on the backbone methine (−C*H*) region (5.78–6.05 ppm). These (−C*H*) protons are denoted as *a* and *b* corresponding to **A3** and **B3** clusters. Followed by this, we denoted backbone methine (−C*H*) protons as *a1*, *a2*, *a3* and *b1*, *b2*, *b3* corresponding to **A2B** and **AB2** clusters (see Figures 1c and 2). The observed distribution patterns were found to closely match those predicted by a binomial probability model derived from Bernoulli trials.^32^ In this model, incorporation of subcomponent **A** or **B** into each of the three coordination sites on the tricopper framework is assumed to occur independently and with a defined probability, determined by the initial molar ratio of the two subcomponents.

**Figure 2:**
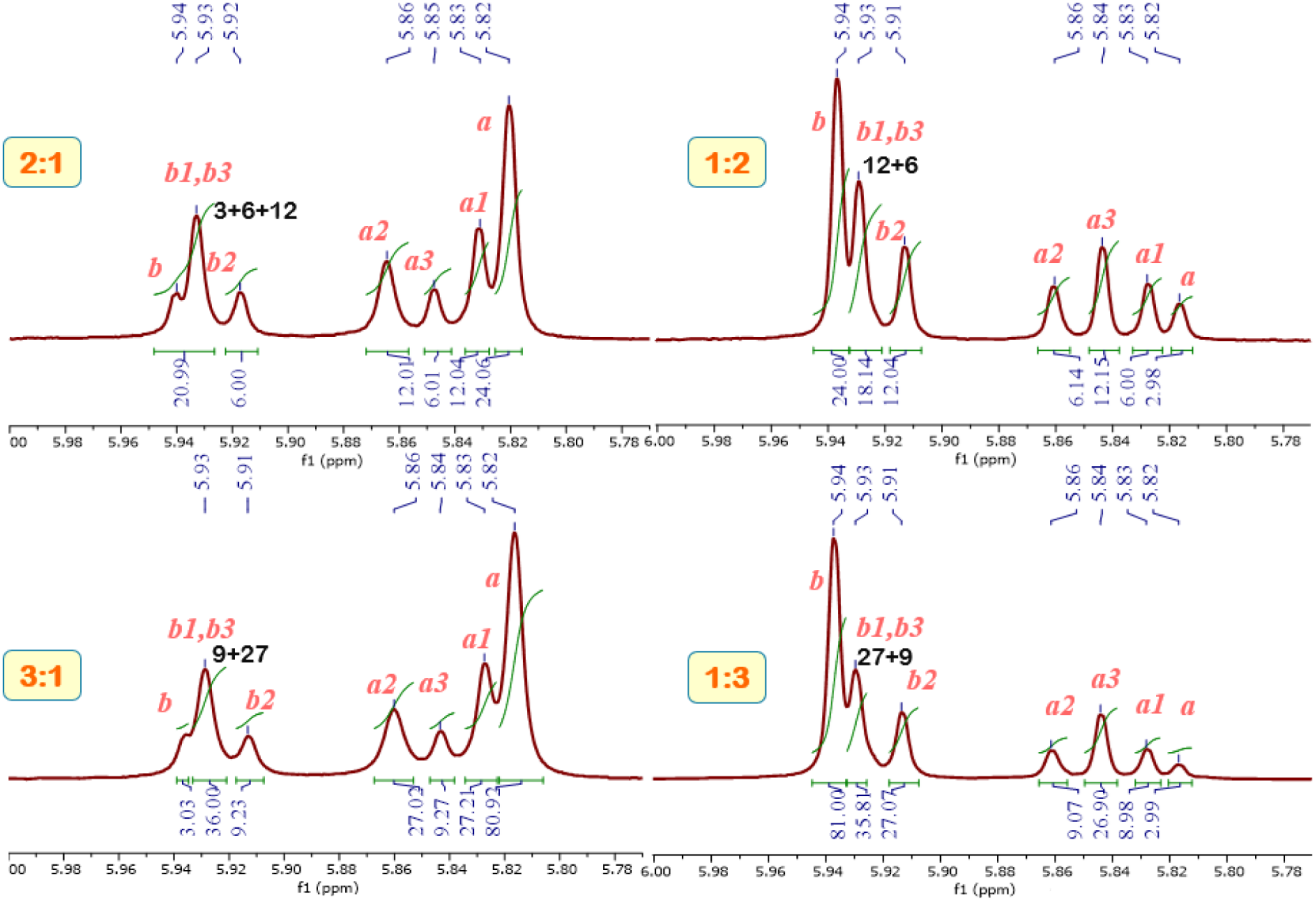
Comparison of subcomponent self-assembly ^1^H NMR results of [**L_A_** Cu]_3_ (**A3**) and [**L_B_** Cu]_3_ (**B3**) (5.77 to 5.97 ppm) in ^1^H NMR with different ratios of **A3**:**B3**, respectively.

To experimentally evaluate this statistical model, scrambling reactions were carried out through two complementary approaches: (i) mixing preformed complexes **A3** to **B3** in varying molar ratios (Path A) and (ii) by *in situ* self-assembly from ligands H**L**_**A**_ and H**L**_**B**_ with two equivalents of CuO^*t*^Bu (Path B), as illustrated in Scheme 3. Reactions were performed using input ratios of 1:1, 1:2, 2:1, 1:3, and 3:1 for **A**:**B** (or H**L**_**A**_:H**L**_**B**_), and the resulting product mixtures (**SP1**) were characterized by ^1^H NMR spectroscopy. The relative amounts of the four main species in (**SP1**): [****L**_**A**_** Cu]_3_ ( **A3** ), [(****L**_**A**_** Cu)_2_(****L**_**B**_** Cu)] ( **A2B** ), [(****L**_**A**_** Cu)­(****L**_**B**_** Cu)_2_] ( **AB2** ), and [****L**_**B**_** Cu]_3_ ( **B3** ), were determined by integrating their methine (−C*H*) signals in the diagnostic spectral region.

The experimental product distributions exhibited excellent agreement with theoretical values predicted from binomial coefficients, as summarized in Table 1. These findings significantly support a statistically governed and stoichiometrically controlled process of subcomponent self-assembly. The successful application of this probabilistic framework highlights the predictive power of combining coordination chemistry with statistical models for understanding dynamic combinatorial systems. To further validate the binomial model, additional scrambling experiments were performed across a broader range of subcomponent input ratios. The resulting ^1^H NMR spectra in the −C*H* region (5.80–6.00 ppm) were analyzed to extract the integral values corresponding to each species (**SP1** components). Representative spectra for the different ratios are shown in Figure 2, with full spectral datasets provided in Figures S1–S5. Across all experiments, the observed product distributions closely matched the predicted statistical outcomes,^33–35^ reinforcing the reliability of the binomial model as a tool for describing self-assembly in these tricopper­(I) complex systems.

**Table 1:** Theoretical Binomial Product Distributions Based on Bernoulli Trials Compared with NMR-Determined Experimental Product Ratios

**[L**_**A**_Cu]_3_:[**L**_**B**_Cu]_3_	Theoretical ratios of SP1^a^	NMR experimental ratios of SP1
1:1	1:3:3:1	1.00 ± 0.4:3.00 ± 0.2:3.00 ± 0.3:1.07 ± 0.5
1:2	1:6:12:8	1.00 ± 0.3:6.11 ± 0.4:12.04 ± 0.5:8.04 ± 0.4
2:1	8:12:6:1	8.00 ± 1.4:12.02 ± 0.5:5.94 ± 1.3:1.02 ± 0.4
1:3	1:9:27:27	1.00 ± 0.5:9.28 ± 0.7:27.05 ± 1.5:27.00 ± 0.9
3:1	27:27:9:1	27.00 ± 1.6:27.13 ± 0.4:9.16 ± 0.4:1.00 ± 0.5

a Scrambled products **SP1** include [**L**_**A**_Cu]_3_, [(**L**_**A**_Cu)_2_(**L**_**B**_Cu)], (**L**_**A**_Cu) (**L**_**B**_Cu)_2_, and [**L**_**B**_Cu]_3_ at equilibrium.

In addition to the dynamic combinatorial formation or self-assembly behavior of copper complexes **SP1** using backbone (−C*H*) methine protons, we observed interesting ^1^H NMR signals corresponding to isopropyl (−C*H*) protons, which result in a set of merged septets. The subcomponents of **SP1**, including **A3**, **A2B**, **AB2**, and **B3**, exhibit dynamic behavior in the solution state. During the ^1^H NMR analysis using a 600 MHz NMR instrument, we observed that the isopropyl signals yielded a complicated spectral pattern between δ 2.88 and δ 3.02, indicating that the dynamic combinatorial behavior could be detected on the NMR timescale. We assigned the isopropyl (−C*H*) protons based on their differences in intensities obtained from stoichiometrically controlled scrambled product formations of **SP1**. In addition, these signals also follow the statistical product distribution, such as a 1:1 ratio of **A3**:**B3** reactants gave the **SP1** products in a ratio of 1:3:3:1 as anticipated earlier. We assigned the 6 isopropyl (−C*H*) protons for **B3** as *f*, the **AB2** contributes 12 isopropyl (−C*H*) proton signals assigned as two sets, *f1* and *f2*, and there are 6 isopropyl (−C*H*) protons from **A2B** assigned as *f3*, as shown in Figure 3.

**Figure 3:**
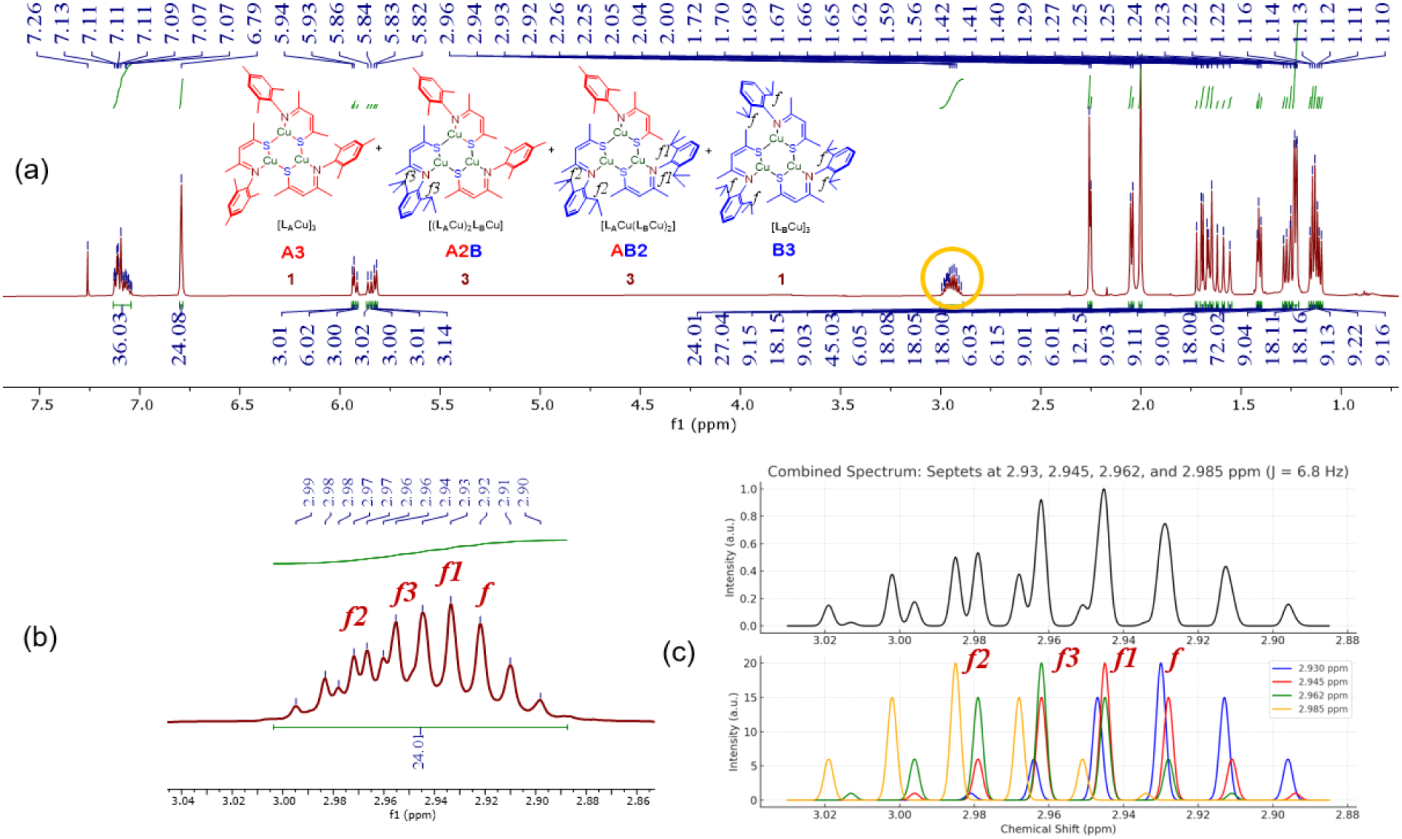
(a) ^1^H NMR of the subcomponent-exchanged product mixture **SP1** (1:1 ratio). (b) Expanded view of the isopropyl (−C*H*) backbone region (2.86 to 3.04 ppm). (c) The septet sets simulation patterns using Matplotlib charts and their assignment of individual isopropyl (−C*H*) protons in the **SP1** mixture, corresponding to the three subcomponents **A2B**, **AB2**, and **B3.**

To gain a better understanding into the complicated ^1^H NMR signaling patterns of the **SP1** mixture, we used Matplotlib to simulate spectral profiles,^36^ which showed excellent agreement with experimental results. Figure 3b,c presents the experimental and simulated ^1^H NMR spectra for the 1:1 ratio **A3**:**B3** mixture, showing excellent agreement. Each isopropyl (−C*H*) septet is color-coded based on its chemical shift: the **B3** septet at 2.930 ppm is depicted in blue (assigned as *f*), the **AB2** septets at 2.945 and 2.985 ppm are shown in red and yellow (*f1* and *f2*, respectively), and the **A2B** septet at 2.962 ppm is represented in green (*f3*). Simulated and experimental septet patterns for additional stoichiometric ratios (1:2, 1:3, 2:1, and 3:1) are provided in Figure S6, further validating the consistency of the dynamic combinatorial behavior across varying **A3**:**B3** ratios.

Furthermore, we estimated the equilibrium constants (*K*) for the formation of the **SP1** mixture under each condition using the integrated intensities of the methine (−C*H*) signals from the ^1^H NMR spectra. Across both synthetic pathways (Scheme 3, Path **A** and Path **B**), the equilibrium constant was found to be approximately *K* ≈9 ± 0.32, indicating a consistent thermodynamic driving force regardless of the assembly route. For non-equimolar feed ratios such as 1:2, 2:1, 1:3, and 3:1, the equilibrium constant *K* remains formally identical; however, the statistical product distribution shifts according to the mole fraction of each precursor, consistent with binomial redistribution behavior. This dynamic equilibrium behavior, observed in both preassembled and *in situ*-assembled systems, underscores the robustness of the self-assembly process. Comparable molar-dependent equilibria have been reported in related dynamic combinatorial systems, notably by Sanders and Otto, in studies involving cinchona alkaloids and ruthenium and iridium trimers.^15,16^ These precedents reinforce the validity of our approach and underscore the utility of *β*-thioketiminato-based tricopper­(I) clusters as platforms for investigating statistical self-assembly. Our findings demonstrate that *β*-thioketiminate-based tricopper­(I) clusters are excellent platforms for studying statistical self-assembly and that product distributions in such systems can be effectively predicted and rationalized using binomial probability models.

### Temperature-Dependent, Thermodynamic Analysis of *β*-Thioketiminate Cu­(I) Trimer Self-Assembly

In order to understand the deeper insights into the thermodynamics of coordination-driven self-assembly in *β*-thioketiminato copper­(I) clusters (**SP1**), temperature-dependent ^1^H NMR studies were performed. These experiments probe the temperature-induced changes or shifts in equilibrium between homoleptic **A3** and **B3** clusters versus heteroleptic clusters **A2B** and **AB2**. The temperature dependence of the equilibrium constant was analyzed using the Van’t Hoff relation (eq 1), to construct the corresponding plots, from which the thermodynamic parameters Δ*H* and Δ*S* for the dynamic redistribution were determined.

1 $$\ln K=-\frac{\Delta H}{R}\left(\frac{1}{T}\right)+\frac{\Delta S}{R}$$

where *K* is the equilibrium constant, Δ*H* is the change in enthalpy, Δ*S* is the change in entropy, and *R* is the gas constant. A linear Van’t Hoff plot (ln *K* vs 1/*T*) indicates temperature-independent enthalpy and entropy, while non-linear behavior suggests more complex thermodynamics, such as changes in heat capacity (Δ*Cp* ≠ 0),^37^ multiple equilibria,^38^ or structural reorganization in the system.^38,39^ In dynamic combinatorial systems, such deviations often reflect temperature-dependent speciation and competing assembly pathways, providing deeper insight beyond simple two-state models.

The equimolar mixture of the homoleptic *β-*thioketiminate copper­(I) trimers [**L**_**A**_Cu]_3_ (**A3**) and [**L**_**B**_Cu]_3_ (**B3**) undergoes rapid subcomponent self-assembly in CD_2_Cl_2_ to establish the dynamic equilibrium shown in eq 2.

A3+B3⇌A2B+AB2 2

where **A2B** and **AB2** represent the heteroleptic mixed-trimer species (Figure 4a). At 298 K, the ^1^H NMR spectrum exhibits well-resolved 24 backbone (−C*H*) methine resonances in the 5.75–6.00 ppm region (Figure 4b and c), corresponding to the distinct **A** subcomponent (*a*, *a1*–*a3*) and **B** subcomponent (*b*, *b1*–*b3*) proton signals in the corresponding to four trimeric clusters. Integration of these signals yields an equilibrium constant $K=\frac{\left[A2B\right]\left[AB2\right]}{\left[A3\right]\left[B3\right]}=9.01$ , which matches the statistical value of 9 expected for a purely random binomial distribution (1:3:3:1 ratio of **A3**:**A2B**:**AB2**:**B3**). This result confirms that rapid redistribution of ligand coordination units **A** and **B** , occurs on the NMR timescale at 298 K, generating a dynamic equilibrium with products distributions closely matches near the binomial statistics under ambient conditions.

**Figure 4:**
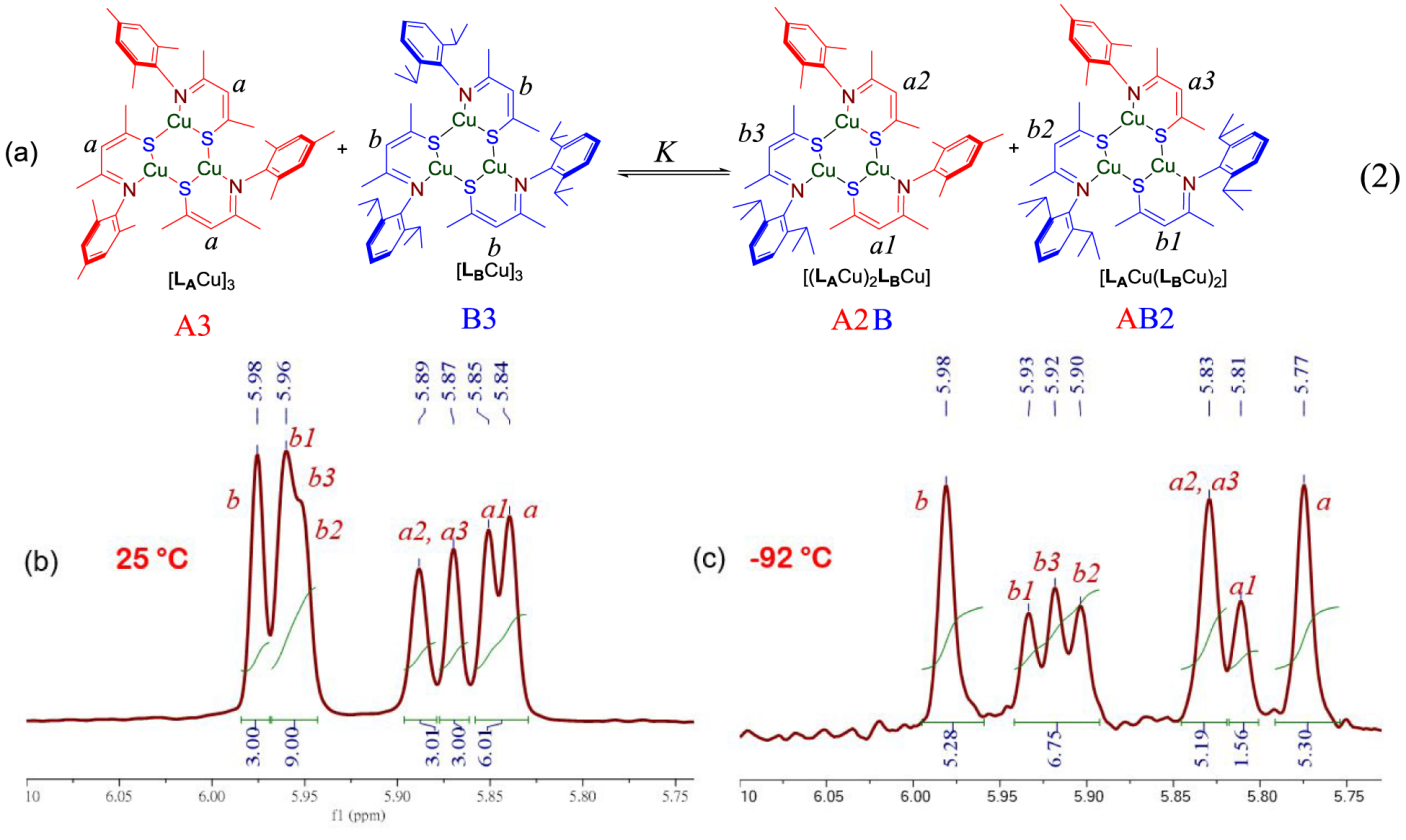
(a) Schematic representation of subcomponent-exchanged product mixture **SP1** (1:1) at equilibrium with the parent trimers, [**L_A_** Cu]_3_ (**A3**) and [**L_B_** Cu]_3_ (**B3**). (b) Expanded view of the methine (−C*H*) backbone region (δ = 5.74–6.00 ppm) of the ^1^H NMR spectrum at 298 K, showing the assignment of individual (−C*H*) proton resonances corresponding to the four major species present in **SP1** (**A3**, **A2B**, **AB2**, and **B3**). (c) Corresponding expanded view of the same region at 181 K, illustrating temperature-dependent spectral changes and enabling clear resolution and assignment of the (−C*H*) proton resonances for all four subcomponent clusters.

Variable-temperature ^1^H NMR spectra recorded from 298 K to 181 K reveal pronounced temperature-dependent changes in both chemical shifts and relative integrals of the methine resonances; see Figures 5a and S7–S9). As the temperature is lowered, the **B**-subcomponent ^1^H signals (*b*, *b1*–*b3*) remain relatively sharp and shift consistently upfield, while the **A**-subcomponent ^1^H signals broaden and merge progressively, averaging a consistent value with a dynamic exchange process that slows on the ^1^H NMR timescale. Quantitative integration of the resolved peaks at each temperature (Table S1) affords the equilibrium constants listed in Table 2 and their detailed calculations shown in Table S2. *K* decreases monotonically from 9.01 at 298 K to 1.63 at 181 K, demonstrating that the equilibrium shifts toward the homoleptic trimers **A3** and **B3** at lower temperatures. The corresponding free-energy changes (Δ*G* = −*RT* ln *K*) become progressively less negative, from −5.45 kJ mol^–1^ (298 K) to −0.63 kJ mol^–1^ (181 K), confirming that heteroleptic assembly is spontaneous but increasingly disfavored upon cooling.

**Figure 5:**
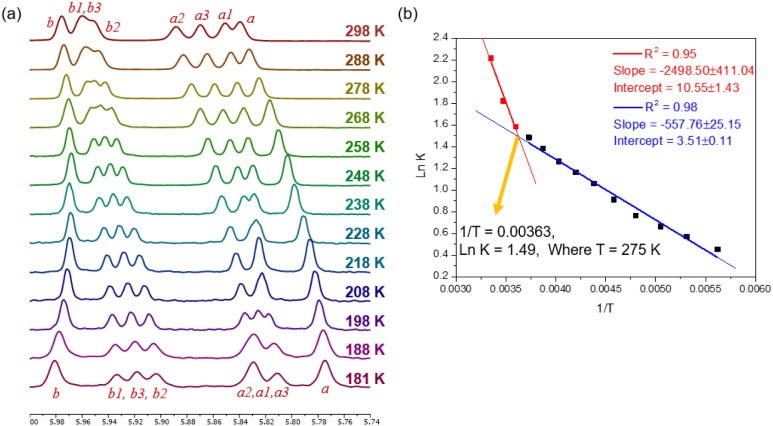
(a) Variable-temperature ^1^H NMR spectra (δ ≈5.74–6.00 ppm) of the methine (−C*H*) region for the (**A3**, **A2B**, **AB2**, and **B3**) **SP1** mixture, showing temperature-dependent evolution, and assignment of the resonances corresponding to Progressive coalescence at higher temperatures indicates rapid, reversible exchange between cluster components. (b) Van’t Hoff plot (ln *K* vs 1/*T*) derived from VT-NMR data, displaying two distinct linear regimes with a crossover near 275 K, indicating a temperature-dependent shift in the dominant equilibrium.

**Table 2:** Temperature-Dependent Thermodynamic Parameters Derived from VT-NMR Data for **A3**/**B3** Self-Assembly, Highlighting Two Distinct Van’t Hoff Regimes

*T* (K)	1/*T* (K^–1^)	*K*	ln *K*	Δ*H* (kJ/mol)	Δ*S* (kJ/mol K)	*T*Δ*S* (kJ/mol)	Δ*G* (kJ/mol)
298	0.00335	9	2.197	20.772	0.088	26.224	–5.452
288	0.00347	6.67	1.898	20.772	0.088	25.344	–4.572
278	0.0036	5.29	1.666	20.772	0.088	24.464	–3.692
268	0.00373	4.35	1.47	4.637	0.0291	7.7988	–3.162
258	0.00387	3.77	1.327	4.637	0.0291	7.5078	–2.871
248	0.00403	3.44	1.235	4.637	0.0291	7.2168	–2.58
238	0.0042	3.09	1.128	4.637	0.0291	6.9258	–2.289
228	0.00438	2.8	1.03	4.637	0.0291	6.6348	–1.998
218	0.00458	2.4	0.875	4.637	0.0291	6.3438	–1.707
208	0.0048	2.09	0.737	4.637	0.0291	6.0528	–1.416
198	0.00505	1.94	0.663	4.637	0.0291	5.7618	–1.125
188	0.00531	1.81	0.593	4.637	0.0291	5.4708	–0.834
181	0.00562	1.63	0.489	4.637	0.0291	5.2671	–0.63

A Van’t Hoff analysis (ln *K* versus 1/*T*) is nonlinear and exhibits a clear inflection near 275.7 K (≈2.5 °C), indicating two distinct thermodynamic regions (Figure 5b). Linear least-squares fitting of the high-temperature region (298–278 K) yields Δ*H* = +20.8 ± 3.4 kJ mol^–1^ and Δ*S* = +0.09 ± 0.012 kJ mol^–1^ K^–1^ (*R* ^2^ = 0.95). The low-temperature region (268–181 K) gives smaller values of Δ*H* = +4.7 ± 0.2 kJ mol^–1^ and Δ*S* = +0.030 kJ mol^–1^ K^–1^ (*R* ^2^ = 0.98). Both regions are characterized by positive enthalpy and positive entropy, consistent with an endothermic yet entropy-driven assembly process. Despite the enthalpic penalty associated with heteroleptic trimer formation, the favorable −*T*Δ*S* term dominates over the entire temperature range, maintaining Δ*G* < 0.

The larger entropic contribution at higher temperatures favors heteroleptic scrambling, resulting in the near-statistical 1:3:3:1 product distribution observed at 298 K. Upon cooling, attenuation of this entropic driving force leads to progressive enrichment of the homoleptic species, with the product ratios evolving toward approximately 1.6:2.5:2.5:1.6 at 181 K, approaching the idealized low-temperature limit of 2:2:2:2. The observed non-linearity in the Van’t Hoff plot suggests that the thermodynamic parameters governing the equilibrium are temperature-dependent. Such behavior is consistent with an entropy-driven dynamic combinatorial system in which configurational entropy arising from ligand/monomer mixing competes with increasingly dominant enthalpic preferences at low temperatures.^40–42^

The positive Δ*H* indicates that heteroleptic assembly is slightly enthalpically disfavored, likely due to subtle steric or electronic mismatches between the dissimilar **A** and **B** ligand-copper units within the trinuclear core, yet this penalty is more than offset by the entropic gain from forming two distinct heteroleptic species from two homoleptic parents at high temperatures. The break near 275 K may reflect a change in the dominant solvation or conformational contributions obtained at a crossover between fast exchange and slow exchange at lower temperature regions that is altered by the effective enthalpic-controlled equilibrium mixture. These findings highlight that temperature provides a simple external stimulus to control the nuclearity and ligand distribution in coordination-driven self-assembly, offering a straightforward route to switch between statistical heteroleptic mixtures and homoleptic-enriched ensembles in supramolecular libraries. Overall, the VT-NMR study demonstrates that the **A3/B3** *β-*thioketiminate Cu­(I) system exemplifies a predominantly entropy-driven, temperature-responsive dynamic equilibrium. The results highlight the utility of VT-NMR-derived Van’t Hoff analyses for quantifying subtle thermodynamic driving forces in multinuclear metal assemblies and provide a quantitative framework for designing stimuli-responsive supramolecular architectures in which ligand self-sorting can be predictably tuned by temperature.

### ESI-MS Characterization of *β-*Thioketiminate Copper­(I) Complexes

In recent years, electrospray ionization mass spectrometry (ESI-MS), as a soft ionization technique, has been used to study the product formations obtained by ligand exchange-based self-assembly or coordination-driven self-assembly reactions or DCLs.^33,43–45^ To complement the NMR studies and further investigate the behavior of *β-*thioketiminato copper­(I) clusters, ESI-MS was employed. However, due to the neutral nature of the [**L**Cu]_3_ complexes, direct detection of intact tricopper­(I) clusters was not feasible under standard ESI conditions; only signals corresponding to the free ligands were observed. To address this limitation, we treated the neutral tricopper­(I) clusters with a minimum of three equivalents of alkali metal salts, such as CsCl, in THF or MeOH. This approach facilitates ionization by generating positively charged adducts through coordination of the soft Cs^+^ cation to the electron-rich cluster framework (Scheme S1). Upon treatment with CsCl, we observed minor but distinct peaks corresponding to Cs^+^-coordinated tricopper­(I) adducts or [Cs@[**L**Cu]_3_]^+^, confirming successful formation of ionizable species suitable for ESI-MS analysis. This cesium-bound species was detected as low-intensity fragment peaks consistent with the expected m/z values, and their identities were further validated through comparison with simulated mass spectra (see Figures S11–S13).

### ESI-MS Analysis of Subcomponent Self-Assembly in Tricopper­(I) Clusters

To further explore the subcomponent self-assembly behavior of *β-*thioketiminato copper­(I) clusters, we extended ESI-MS characterization to mixtures of [**L**_**A**_Cu]_3_ (**A3**) and [**L**_**B**_Cu]_3_ (**B3**). These mixtures, previously shown by ^1^H NMR to generate statistical scrambling products (**SP1**) with binomial distributions, were treated with CsCl to facilitate detection *via* ESI-MS. A 1:1 mixture of **A3** and **B3** with three equivalents of CsCl or CsOAc was expected to yield a family of cesium-coordinated cluster adducts (**Cs@SP1**), reflecting the underlying self-assembled product distribution (Scheme 4). Interestingly, while the overall product pattern observed in ESI-MS mirrored that of the NMR experiments, the relative intensities of the **Cs@SP1** species deviated from the theoretical statistical distributions. Specifically, for the 1:1 **A3**:**B3** mixture, the product ratio derived from ESI-MS intensities was approximately 1:2:2:1, compared to the expected 1:3:3:1 distribution from the binomial model (Figure 6a). Further, we examined the influence of stoichiometry. We performed a series of ESI-MS experiments using varying input ratios of **A3** and **B3** (1:1, 1:2, 1:3, 2:1, and 3:1), each with stoichiometric amounts of CsCl. The resulting **Cs@SP1** product distributions, as determined by ESI-MS spectral data, showing the relative intensities of the corresponding peaks, are displayed in Figure 6b, with quantitative summaries provided in Table 3. Full spectral data, along with simulated mass spectra for 1:1 ratio validation, are available in Figure S13. The observed deviations in product distribution suggest that while ESI-MS can qualitatively reflect the diversity of self-assembled species, quantitative analysis may be affected by differences in ionization efficiency, the stability of adducts in the gas phase, or the inherent complexity of multinuclear systems. Nevertheless, the consistency between experimental and simulated fragmentation patterns supports the structural assignments of the **Cs@SP1** adducts and confirms the dynamic, self-assembling nature of these tricopper­(I) systems under both neutral and ionizing conditions.

**Scheme 4:**
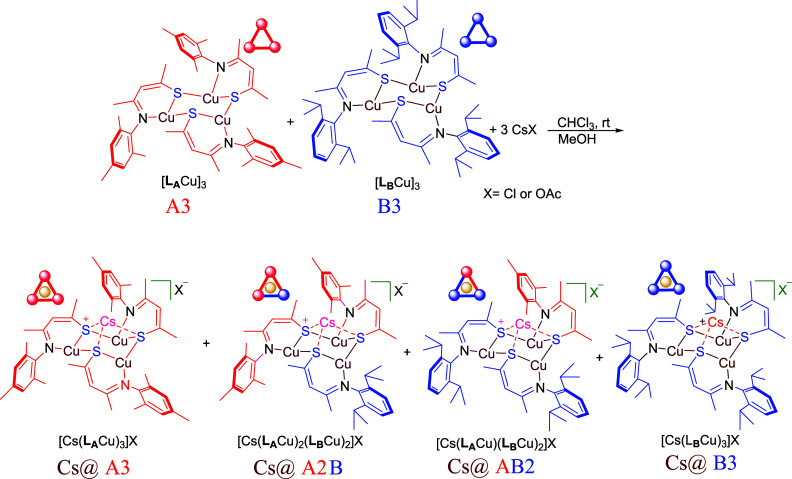
Schematic Representation of Cesium-Assisted Self-Assembly of *β-*Thioketiminato copper­(I) Clusters^a^

a Mixing [**L**_**A**_Cu]­3 (**A3**) and [**L**_**B**_Cu]­3 (**B3**) in a 1:1 molar ratio in the presence of CsCl or CsOAc yields Cs^+^-coordinated self-assembled products, as detected by ESI-MS.

**Table 3:** Theoretical Binomial Product Distributions Based on Bernoulli Trials Compared with ESI-MS-Determined Experimental Product Ratios

**[L**_**A**_Cu]_3_:[**L**_**B**_Cu]_3_	Theoretical ratios of **SP1** ^a^	ESI-MS experimental ratios of **Cs@SP1**
1:1	1:3:3:1	1.21 ± 0.4:2.11 ± 1.7:2.22 ± 1.4:1.11 ± 0.5
1:2	1:6:12:8	1.65 ± 1.6:3.11 ± 3.2:7.33 ± 6.4:11.11 ± 3.4
2:1	8:12:6:1	6.52 ± 2.7:7.70 ± 5.6:3.70 ± 2.4:1.76 ± 1.4
1:3	1:9:27:27	1.75 ± 1.2:3.47 ± 0.4:6.65 ± 0.4:7.70 ± 0.4
3:1	27:27:9:1	10.89 ± 11.4:9.65 ± 6.4:2.50 ± 0.4:1.57 ± 0.4

a Scrambled products **Cs@SP1** include **Cs@**[**L**_**A**_Cu]_3_, **Cs@**[(**L**_**A**_Cu)­(**L**_**B**_Cu)_2_], **Cs@**(**L**_**A**_Cu) (**L**_**B**_Cu)_2_, and **Cs@**[**L**_**B**_Cu]_3_ at equilibrium.

**Figure 6:**
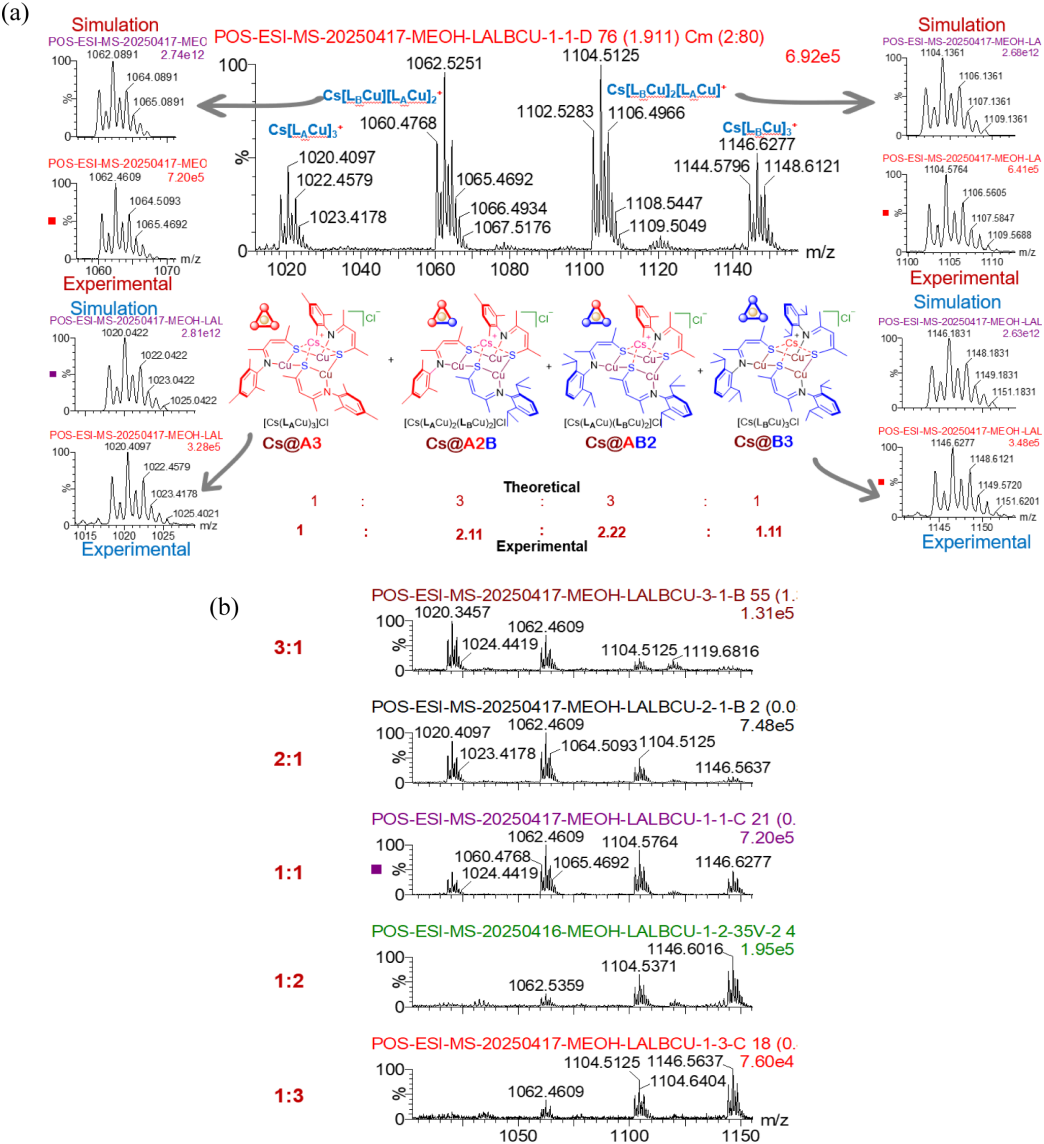
(a) ESI-MS spectral interpretation of cesium-coordinated self-assembled tricopper­(I) clusters. (b) Product distribution analysis of **SP1** species obtained from mixtures of **A3** and **B3** in varying molar ratios, based on ESI-MS peak intensities.

### ^133^Cs NMR as a Probe for Examination of Subcomponent Self-Assembly in Tricopper­(I) Clusters

To investigate the dynamics of cesium coordination in the adducts **Cs@[LCu]**_**3**_ and **Cs@SP1**, we recorded ^133^Cs NMR spectra using CsOAc as the cesium source in CH_2_Cl_2_/CH_3_OH mixtures with **A3**, **B3**, and **SP1**. These results reveal that they undergo fast exchange based on the NMR timescale.^46,47^ Titration experiments show weak-to-moderate upfield chemical shift changes upon incremental addition of CsOAc, consistent with reversible [**L**Cu]_3_-Cs^+^ interactions (see Figures S11–S13). The observed ^133^Cs resonances for **Cs@A3**, **Cs@B3**, and **Cs@SP1** are available in Table S3. The ^133^Cs NMR spectra display a single resonance that evolves smoothly with Cs^+^ concentration, reflecting rapid exchange of the guest among the available hosts in solution. Under these conditions, the Cs^+^ nucleus provided a time-averaged electronic environment of the tricopper cavity rather than resolving individual host identities. Consequently, while the heteroleptic **Cs@SP1** framework follows the same overall chemical-shift trend as the homoleptic analogues **Cs@A3** and **Cs@B3**, its resonance appears slightly upfield, consistent with subtle differences in the electronic environment arising from ligand asymmetry rather than changes in host speciation probing averaged host–guest electronic environments under fast exchange.

### Solvation Effects and DFT Investigations on the Thermodynamic Origins of Subcomponent Scrambling

The solvent interactions can influence the stability and pathway accessibility of labile Cu­(I) assemblies, and we further explored the solvent dependence of the scrambling process to gain molecular-level insight into the temperature-dependent redistribution behavior observed by VT-NMR. However, substantial differences in cluster solubility were observed across a broad range of nonhalogenated solvents, including protic, polar aprotic, and nonpolar media (MeOH, EtOH, CH_3_CN, THF, acetone, toluene, and *n*-hexane). **A3** rapidly precipitated, possibly because of limited solubility or an increased tendency toward aggregation, whereas **B3** remained soluble. Consequently, homogeneous redistribution equilibria could not be established, highlighting the critical role of halogenated solvents in maintaining homogeneous solutions in which scrambling processes can be observed.

To gain qualitative molecular-level insights into experimentally observed temperature-dependent scrambling behavior, DFT calculations were employed. We performed these calculations at the B3LYP-D3/def2-TZVP (Cu) and 6-311G** (ligand atoms) levels of theory using the SMD solvation model with CH_2_Cl_2_ to approximate the experimentally relevant solution environment. The experimentally derived free-energy differences are small and highly temperature-dependent, and the analysis focuses on qualitative trends in pathway accessibility and possible redistribution pathways rather than establishment of a definitive mechanistic model.

Although associative exchange pathways cannot be excluded, attempts to locate stable close-contact associative aggregates of two tricopper clusters were unsuccessful. Consequently, our focus shifted to partially dissociated and CH_2_Cl_2_-associated configurations, which correspond to identifiable minima on the computed potential energy surface and provide tractable models for examining qualitative trends in pathway accessibility. The DFT-identified minima suggest that, under halogenated solvent conditions, homoleptic trimers **A3** and **B3** can access configurations that enable redistribution into heteroleptic **A2B** and **AB2** clusters, as summarized in Scheme 5.

**Scheme 5:**
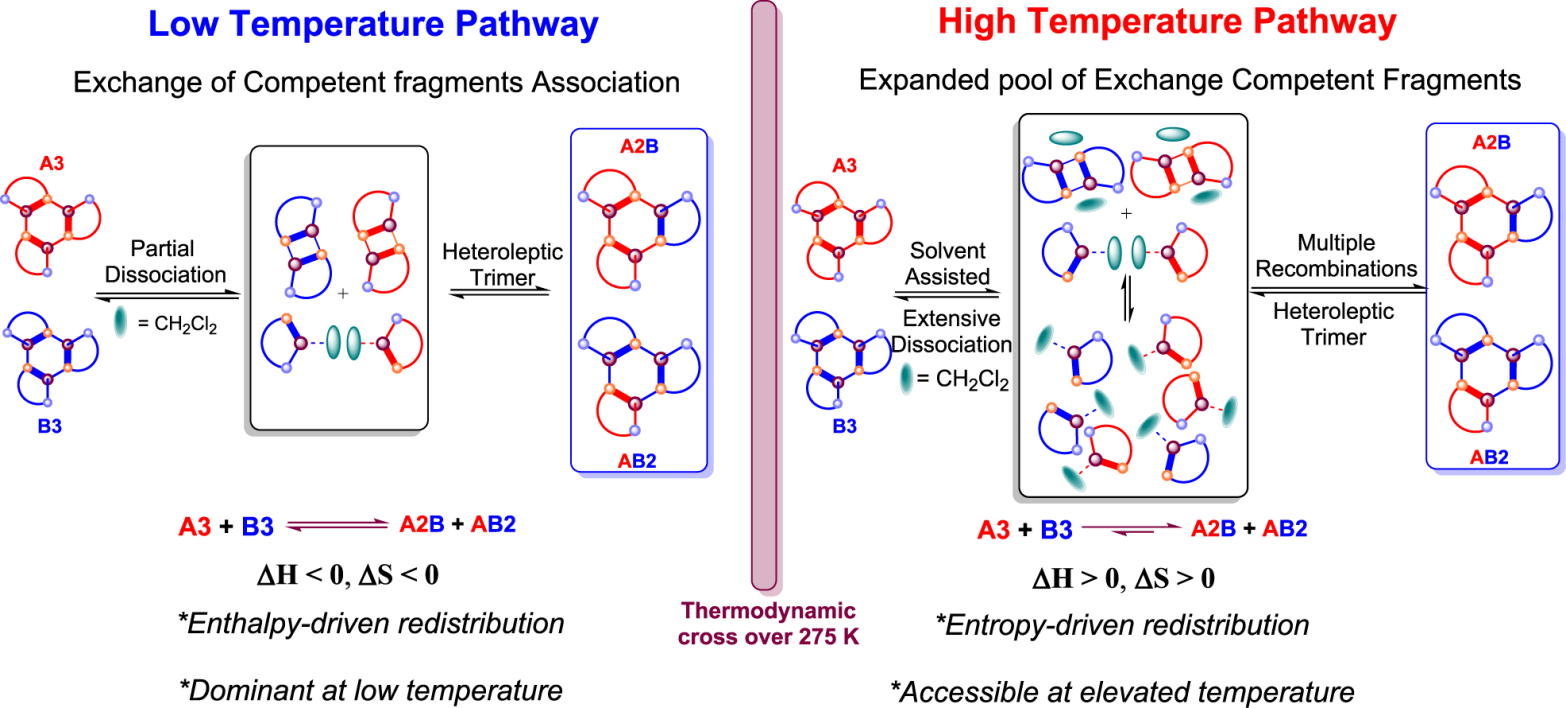
Possible Temperature-Dependent Ligand Scrambling Routes in *β-*Thioketiminato tricopper­(I) Clusters

Two thermodynamic regimes may account for the experimentally observed behavior. (i) Low-temperature regime: Redistribution is consistent with partial dissociation followed by exchange and recombination of a relatively limited pool of accessible fragments. The overall process is characterized by favorable enthalpy (Δ*H* < 0) but negative entropy (Δ*S* < 0), consistent with enthalpy-driven redistribution that becomes increasingly important upon cooling. (ii) High-temperature regime: In addition to the low-temperature redistribution processes, more extensive CH_2_Cl_2_-associated dissociation becomes thermally accessible at elevated temperatures, leading to an expanded pool of exchange-accessible fragments and additional recombination possibilities. The overall process is associated with positive entropy (Δ*S* > 0) and provides a qualitative thermodynamic rationale for the enhanced scrambling and near-statistical product distributions observed at higher temperatures.

The experimentally observed crossover near 275 K in the Van’t Hoff plot thus reflects a transition between distinct redistribution mechanisms: enthalpy-dominated exchange via limited intermediates at low temperatures versus entropy-enhanced scrambling through a broader ensemble of solvated fragments at elevated temperatures. Configurational entropy estimates for the low- and high-temperature ensembles align reasonably with the experimentally derived Δ*S* values from VT-NMR, with additional contributions arising from conformational flexibility and solvent reorganization. Full details of all elementary steps (Scheme S2), optimized structures (Figures S17–S19), individual Δ*H*/Δ*S* values, and extended entropy calculations (Tables S4-S7) are provided in the Supporting Information.

## Conclusions

This study establishes the dynamic combinatorial self-assembly behavior of *β-*thioketiminato tricopper­(I) clusters, [**L**_**A**_Cu]_3_ (**A3**) and [**L**_**B**_Cu]_3_ (**B3**), through a combined experimental and computational investigation. ^1^H NMR analyses demonstrate that mixing the two homoleptic trimers generates a well-defined ensemble of homo- and heteroleptic species (**A3**, **A2B**, **AB2**, and **B3**), whose distributions closely follow binomial statistics. These observations indicate that the scrambling process proceeds through reversible redistribution of preorganized Cu–ligand coordination units rather than complete ligand dissociation, emphasizing the dynamic yet structurally persistent nature of the Cu­(I) cluster framework and the intrinsic lability of Cu–S interactions.

Variable-temperature ^1^H NMR experiments reveal that the dynamic equilibrium is highly temperature-dependent and evolves across two distinct thermodynamic regimes separated by a crossover region near 275 K. At elevated temperatures, the equilibrium approaches near-statistical distributions dominated by favorable configurational entropy arising from increased ensemble diversity. In contrast, lower temperatures favor enthalpy-biased redistribution pathways, resulting in measurable deviations from ideal statistical populations and preferential stabilization of homoleptic assemblies. The resulting non-linear Van’t Hoff behavior therefore reflects a temperature-dependent shift in the dominant scrambling mechanism rather than a single thermodynamic process.

DFT calculations indicate that the observed thermodynamic crossover arises from temperature-dependent access to CH_2_Cl_2_-associated partially dissociated states. At low temperatures, recombination is enthalpy-biased, favoring the formation of trimers from these accessible fragments. At elevated temperatures, additional CH_2_Cl_2_-mediated dissociation becomes thermodynamically accessible, expanding the ensemble of solvated fragments and providing a plausible explanation for entropy-driven scrambling found to be consistent with the qualitative DFT calculations.

ESI-MS independently confirms the statistical **A3/A2B/AB2/B3** distribution observed by VT-NMR, validating the coexistence of multiple equilibrating species in solution. Collectively, these results establish *β-*thioketiminato tricopper­(I) assemblies as model systems for ensemble thermodynamics in multinuclear coordination chemistry, highlighting the interplay between configurational entropy, solvent effects, and temperature-dependent pathway accessibility.

## Experimental Section

All experiments, including the synthesis of CuO^*t*^Bu and *β-*thioketiminato ligands, were conducted following published procedures^30,48^ and under a purified dinitrogen atmosphere inside the glovebox or in vacuo using standard Schlenk line techniques. The ligand synthesis procedures and their spectroscopic characterization are described in the appendix. Chemical reagents were purchased from Sigma-Aldrich Chemical Co. Ltd., Lancaster Chemicals Ltd., or Fluka Ltd. All reagents were used without any further purification. Solvents were dried over Na (toluene and THF) or CaH_2_ (CH_2_Cl_2_, CH_3_CN) and then thoroughly degassed with N_2_ before use. FT-IR spectra were recorded using a Bruker Optics FTIR Alpha OPUS. UV–vis spectra were recorded on an Agilent 8453 spectrometer adapted with a Unisoku USP-203 Cryostat. Elemental analyses were performed using a Heraeus CHN-OS Rapid Elemental Analyzer. ^1^H NMR, ^13^C NMR, and ^133^Cs NMR spectra were recorded using Varian Gemini-600 proton and JEOL JNM ECS FT 400 MHz NMR instruments. Variable-temperature ^1^H NMR experiments were performed using equimolar **A3** and **B3** solutions in CDCl_3_ or CD_2_Cl_2_ solvents with ferrocene as an internal standard at a concentration equivalent to an **A** or **B** unit. Measurements were conducted over a temperature range with 10 °C decrements, employing a liquid nitrogen Dewar to ensure precise temperature control. ESI-MS spectra were collected on a Waters ZQ 4000 mass spectrometer.

### Computational Details

Density functional theory (DFT) calculations were performed using Gaussian 16.^49^ The B3LYP functional with Grimme’s D3 dispersion correction was employed for all calculations.^50–52^ The def2-TZVP basis set was used for Cu atoms,^53^ whereas all remaining atoms were treated using the 6-311G** basis set.^54,55^ Solvent effects of dichloromethane were incorporated using the SMD solvation model.^56^ All optimized structures were confirmed as true minima by the absence of imaginary frequencies. Gibbs free energies at 298 K and 1 atm were obtained from the electronic energies including solvent, zero-point energy, and thermal corrections. To account for the known overestimation of translational entropy in solution-phase calculations, solvation entropy contributions were approximated as 2/3 of their gas-phase values.^57–60^ Temperature-dependent free energies at lower temperatures were subsequently estimated using the calculated enthalpy and entropy terms according to Δ*G* = Δ*H* – *T*Δ*S*.

### General Procedure for the Synthesis of Tricopper­(I) Complexes

#### Preparation of *β-*Thioketiminato tricopper­(I) Clusters

The synthesis of [**L**_**A**_Cu]_3_ and [**L**_**B**_Cu]_3_ complexes was detailed in our previous publications,^27,28^ where they were characterized by using various spectroscopic techniques. Further experimental details concerning the formation and characterization of scrambled product **SP1** are provided below.

#### NMR Sample Preparation for Subcomponent Self-Assembly Studies

##### 1:1 Molar Ratio (Path A):

Under an inert atmosphere, a 25 mL Schlenk tube was charged with a solution of [**L**_**A**_Cu]_3_ (87.5 mg, 98.6 μmol) in 2.5 mL of CH_2_Cl_2_. A separate solution of [**L**_**B**_Cu]_3_ (100.0 mg, 98.6 μmol, in 2.5 mL of CH_2_Cl_2_) was added dropwise to the [**L**_**A**_Cu]_3_ solution. No appreciable color change was observed; the solution remained bright reddish-orange. After stirring for 10 minutes, the solvent was removed under vacuum, and the resulting mixture was characterized by ^1^H and ^13^C NMR, as well as ESI-MS techniques.

##### 1:1 Molar Ratio (Path B):

A mixture of ligand H**L**_**A**_ (100.0 mg, 43.0 mmol) and H**L**_**B**_ (117.0 mg, 43.0 mmol) was dissolved in dichloromethane or THF. This solution was added dropwise to a stirred suspension of CuO^*t*^Bu (123.0 mg, 90.0 mmol) in a 100 mL Schlenk flask. The mixture was stirred for 2 h under an inert atmosphere. The solvent was removed under reduced pressure, and the residue was washed with cold *n*-hexane (2 × 5 mL). The resulting product was characterized by ^1^H NMR, which showed signals corresponding to aromatic, methine, methyl, and isopropyl proton environments, consistent with a scrambled mixture of subcomponent-exchanged species. ^1^H NMR (CDCl_3_, 400 MHz, 298 K, δ): 7.09 (m, 36H, Ar*H*(^*i*^pr)_2_), 6.79 (s, 24H, Ar*H*(Me)_3_), 5.94–5.81 (multi-singlets, 8H, backbone-C*H*), 3.00–2.88 (multiple septet, 24H, C*H*(CH_3_)_2_), 2.26 (d, 36H, *J* = 4 Hz, backbone-C*H*_3_), 2.04 (d, 36H, backbone-C*H*_3_), 2.00 (s, 36H, backbone-C*H*_3_), 1.72–1.55 (multi-singlets, 82H, Ar-C*H*_3_), 1.40 (t, *J* = 4.0 Hz, 27H, CH­(C*H*_3_)_2_), 1.28–1.21 (multi-doublet, 108H, CH­(C*H*_3_)_2_), 1.16–1.09 (multi-doublet, 72H, CH­(C*H*_3_)_2_).

### Varying Molar Ratios:

Following the procedure described for the 1:1 mixture, reactions were conducted with different molar ratios of [**L**_**A**_Cu]_3_ to [**L**_**B**_Cu]_3_:

**2:1 Ratio:** [**L**_**A**_Cu]_3_ (175.0 mg, 197.2 μmol), [**L**_**B**_Cu]_3_ (100.0 mg, 98.6 μmol)

**3:1 Ratio:** [**L**_**A**_Cu]_3_ (262.5 mg, 295.8 μmol), [**L**_**B**_Cu]_3_ (100.0 mg, 98.6 μmol)

**1:2 Ratio:** [**L**_**A**_Cu]_3_ (87.5 mg, 98.6 μmol), [**L**_**B**_Cu]_3_ (200.0 mg, 197.2 μmol)

**1:3 Ratio:** [**L**_**A**_Cu]_3_ (87.5 mg, 98.6 μmol), [**L**_**B**_Cu]_3_ (300.0 mg, 295.8 μmol)

In all cases, the [**L**_**B**_Cu]_3_ solution was added dropwise to the [**L**_**A**_Cu]_3_ solution in CH_2_Cl_2_. No significant color change was observed, and each solution remained bright reddish-orange before NMR characterization.

### ESI-MS Sample Preparation for Cs^+^/Na^+^ Coordinated Tricopper­(I) Cluster Adducts

A tricopper complex ([**L**_**A**_Cu]_3_ or [**L**_**B**_Cu]_3_, 10 mg, ∼10.0 μmol) was dissolved in THF or CHCl_3_ in a Schlenk tube. To this, a solution of CsCl (30.0 μmol in 0.5–1.0 mL of MeOH) was added dropwise under an inert atmosphere. A white cloudy suspension was observed in the bright orange solution, which was stirred for 2 h. The reaction mixture was then filtered and diluted to ∼10^–5^ M in MeOH or MeCN and analyzed by ESI-MS. The remaining crude was concentrated and characterized by NMR. [**Cs@A3**]^+^ or [C_42_H_54_CsCu_3_N_3_S_3_]^+^: calculated 1020.64, found 1020.04, [**Cs@B3**]^+^ or [C_51_H_72_CsCu_3_N_3_S_3_]^+^: calculated 1146.88, found 1146.19.

### ESI-MS Sample Preparation for Subcomponent Self-Assembly Studies

A solution of [**L**_**A**_Cu]_3_ (8.8 mg, ∼10.0 μmol) was prepared in a Schlenk tube by dissolving the complex in 3 mL of dry THF or CHCl_3_. An equimolar solution of [**L**_**B**_Cu]_3_ (10.0 mg, ∼10.0 μmol) in the same solvent was added dropwise under stirring. The resulting mixture remained orange and transparent. Subsequently, a methanolic solution of CsCl (30.0 μmol in 0.5 mL of MeOH) was added, immediately producing a fine suspension. The mixture was stirred at room temperature for 2 h to allow equilibration. For ESI-MS analysis, an aliquot of the reaction mixture was diluted with MeOH or MeCN to a final concentration of ∼10^–5^ M and directly infused. The molecular weights of the **Cs@SP1** mixture containing [**Cs@A3**]^+^, [**Cs@A2B**]^+^, [**Cs@AB2**]^+^, and [**Cs@B3**]^+^ remained constant based on molar dependence; however, the relative intensities of the obtained ESI-MS fragments were significantly variable. ESI-MS: [**Cs@A3**]^+^ or [C_42_H_54_CsCu_3_N_3_S_3_]^+^: calculated 1020.64, found 1020.04, [**Cs@A2B**]^+^ or [C_45_H_60_CsCu_3_N_3_S_3_]^+^: calculated 1062.72, found 1062.11, [**Cs@AB2**]^+^ or [C_48_H_66_CsCu_3_N_3_S_3_]^+^: calculated 1104.80, found 1104.12, [**Cs@B3**]^+^ or [C_51_H_72_CsCu_3_N_3_S_3_]^+^: calculated 1146.88, found 1146.19.

### General Procedure for the ^133^Cs NMR Titration Experiments

Separate 5 mM solutions of the trinuclear copper complexes [**L**_**A**_Cu]_3_, **A3**, or [**L**_**B**_Cu]_3_, **B3**, or the mixture of **A3** and **B3** were prepared in dichloromethane (DCM). Aliquots of each solution were transferred to individual glass vials, and cesium acetate (CsOAc) stock solutions in methanol (MeOH) were added to afford final CsOAc loadings of 1, 2, 4, 6, and 8 equivalents relative to each copper complex. In all cases, the total volume was maintained at 0.60 mL.

The resulting mixtures were stirred at room temperature for 1 hour, after which the solutions were transferred to standard 5 mm NMR tubes. ^133^Cs NMR spectra were recorded at an ambient temperature. Chemical shifts were referenced to CsOAc in MeOH as an internal standard with the ^133^Cs resonance set to 0.0 ppm. Changes in the ^133^Cs chemical shift corresponding to **Cs@**[**L**Cu]_3_ or **Cs@SP1** formations were monitored as a function of CsOAc equivalents (see Figures S11–S13).

## Supplementary Material



## Data Availability

The data supporting this article have been included as part of the supplementary information (SI).

## Notes

Theoretical approach on binomial probability experiments, ^1^H NMR results, variable-temperature ^1^H NMR results, ESI-MS results, ^133^Cs NMR titration results, and DFT computational studies for **A3** and **B3** coordination-driven self-assembly (PDF)
