# Supplementary material for: Coordination-Driven Scrambling in Tricopper(I) Clusters: Thermodynamic Insights into Dynamic Assembly
Source: Inorg Chem. 2026 Jul 25;65(31):18209–23. doi: 10.1021/acs.inorgchem.6c02605 (PMC13463546; doi:10.1021/acs.inorgchem.6c02605)
Supplement: Supplementary file 1 [file ic6c02605_si_001.pdf]

Supporting Information

Coordination-Driven Scrambling in Tricopper(I) Clusters: Thermodynamic Insights into Dynamic Assembly

Venkata Sai Sashankh Penki,[a], *Najeeb Ullah,*[a] *Sri Sudewi,*[bc] *Rahimeh Eshaghi Malekshah,*[a] *Hsing Yin Chen,*[a] *Ya-Fan Lin,*[d*] *and Sodio C.N. Hsu*[ae*]

Corresponding authors: yafan@gms.ndhu.edu.tw (Ya-Fan Lin*), sodiohsu@kmu.edu.tw (Sodio C.N. Hsu*)

[a] Department of Medicinal and Applied Chemistry, Kaohsiung Medical University, Kaohsiung 80708, Taiwan.

[b] Department of Chemistry Education, Faculty of Mathematics and Natural Sciences, Yogyakarta State University, Yogyakarta, 55281, Indonesia

[c] Department of Pharmacy, Faculty of Mathematics and Natural Science, Universitas Sam Ratulangi, Manado 95115, Indonesia

[d] Department of Chemistry, National Dong Hwa University, Hualien 974301, Taiwan

[e] Department of Medical Research, Kaohsiung Medical University Hospital, Kaohsiung 80708, Taiwan

Table of Contents

Theoretical Approach on Binomial Probability Experiments……………………………………S3

^{1}H NMR results ………...……………………………………………………………..…..…..S4

Variable Temperature ^{1}H NMR Results…………………………………….……………..….S11

ESI-MS results………………………………….…………………………….……….…....…S17

[133]Cs NMR Titration results……………………………..……………….….……..….…..S20

DFT Computational Studies for **A3B3** Coordination Driven Self-Assembly……….……….S26

References……………………………………….………………………………….…………..S31

Theoretical Approach on Binomial Probability Experiments

In order to understand the driving force involved in the **SP1** products formation in a well-defined product distribution ratio as per Bernoulli trials, we investigated further using several experiments using stoichiometric strategies. Binomial probability experiments are theoretically obtained by the combination of Bernoulli trials and the binomial probability distribution formula. This formula is defined as follows: If the probability for success in a single trial is represented as P_E, the probability of obtaining all successes represented by j in a binomial experiment of n trials is then the total probability is defined by $(P_E)^j(1-P_E)^{n-j}$ where $(1-P_E)^{n-j}$ represents the probability of $n-j$ unsuccessful events among n trials. The probability of observing a specific order of trial outcomes (i.e., a single permutation) corresponding to j successful trials is then considered. This is followed by $C(n, j)$, which accounts for all possible permutations corresponding to j successful trials.[1] On the other hand, if we change the ratios starting from 1:1 to higher orders then the theoretical product ratios also will change in a specific manner summarized in table 1.

$$P(j) = C(n, j)\,(P_E)^j(1 - P_E)^{n-j}$$

In scrambling product formation of **SP1** (Scheme 3), two trimer complexes, [$\mathbf{L_A}$Cu]$_3$ (**A3**) and [$\mathbf{L_B}$Cu]$_3$ (**B3**), were aggregated and reassembled together to obtain products **A3**, **A2B**, **AB2**, and **B3**. We assume these new **SP1** products are the clusters composed of trimeric structures based on their thermodynamic stability and their equilibrium coexistence. Excluding other possible products (monomer, dimer, tetramer, or polymeric clusters), if we consider the total possible outcome of all trimer structures, where the number of trials n and the number of obtaining each trimer structure j are both 3, the possible outcome of products is defined by the binomial probability distribution formula is given as follows:

- $P(A_3) = C\,(3,3)\,(\frac{1}{2})^{3-0}\,(\frac{1}{2})^{3-3} = 1 \times \frac{1}{8} \times 1 = \frac{1}{8}$
- $P(A_2B) = C\,(3,2)\,(\frac{1}{2})^{3-1}\,(\frac{1}{2})^{3-2} = 3 \times \frac{1}{4} \times \frac{1}{2} = \frac{3}{8}$
- $P(AB_2) = C\,(3,1)\,(\frac{1}{2})^{3-2}\,(\frac{1}{2})^{3-1} = 3 \times \frac{1}{2} \times \frac{1}{4} = \frac{3}{8}$
- $P(B_3) = C\,(3,0)\,(\frac{1}{2})^{3-3}\,(\frac{1}{2})^{3-0} = 1 \times 1 \times \frac{1}{8} = \frac{1}{8}$

From the above ratio calculations of each trimer structure, we can define the possible outcome of the total products distribution included in the **SP1** structure, given by

- $A3\text{: }A2B\text{: }AB2\text{: }B3 = \frac{1}{8}\text{: }\frac{3}{8}\text{: }\frac{3}{8}\text{: }\frac{1}{8} \rightarrow 1\text{: }3\text{: }3\text{: }1 \rightarrow \underline{1+3+3+1}$ = Total 8 different protons we assigned for the –C*H* backbone positions.

Calculation of equilibrium constants for different molar ratios of A3B3 self assembly by ^{1}H NMR analysis of Figures 1c and 2

$$K_{\mathbf{A3:B3}} = \frac{[\mathbf{A2B}][\mathbf{AB2}]}{[\mathbf{A3}][\mathbf{B3}]} = \frac{[a1+a3+b3][b1+b2+a2]}{[3a][3b]}$$

$$K_{1:1} = \frac{[3.00+3.00+3.03][3.03+3.00+3.00]}{[3.00][3.05]} = \frac{[9.03][9.03]}{[3.00][3.05]} = \frac{81.54}{9.15} = 8.91$$

$$K_{2:1} = \frac{[12.04+12.01+12.00][6.00+6.00+6.01]}{[24.06][2.99]} = \frac{[36.05][18.01]}{[24.06][2.99]} = \frac{649.26}{71.94} = 9.02$$

$$K_{1:2} = \frac{[6.00+6.14+6.07][12.07+12.04+12.15]}{[24.00][2.98]} = \frac{[18.21][36.26]}{[24.00][2.98]} = \frac{660.3}{71.52} = 9.23$$

$$K_{3:1} = \frac{[27.21+27.02+27.00][9.00+9.23+9.27]}{[80.92][3.03]} = \frac{[81.23][27.50]}{[80.92][3.03]} = \frac{2233.82}{245.18} = 9.11$$

$$K_{1:3} = \frac{[8.98+9.07+8.90][26.9+27.07+26.91]}{[81.00][2.99]} = \frac{[26.95][80.88]}{[81.00][2.99]} = \frac{2179.71}{242.19} = 9.00$$

^{1}H NMR Results

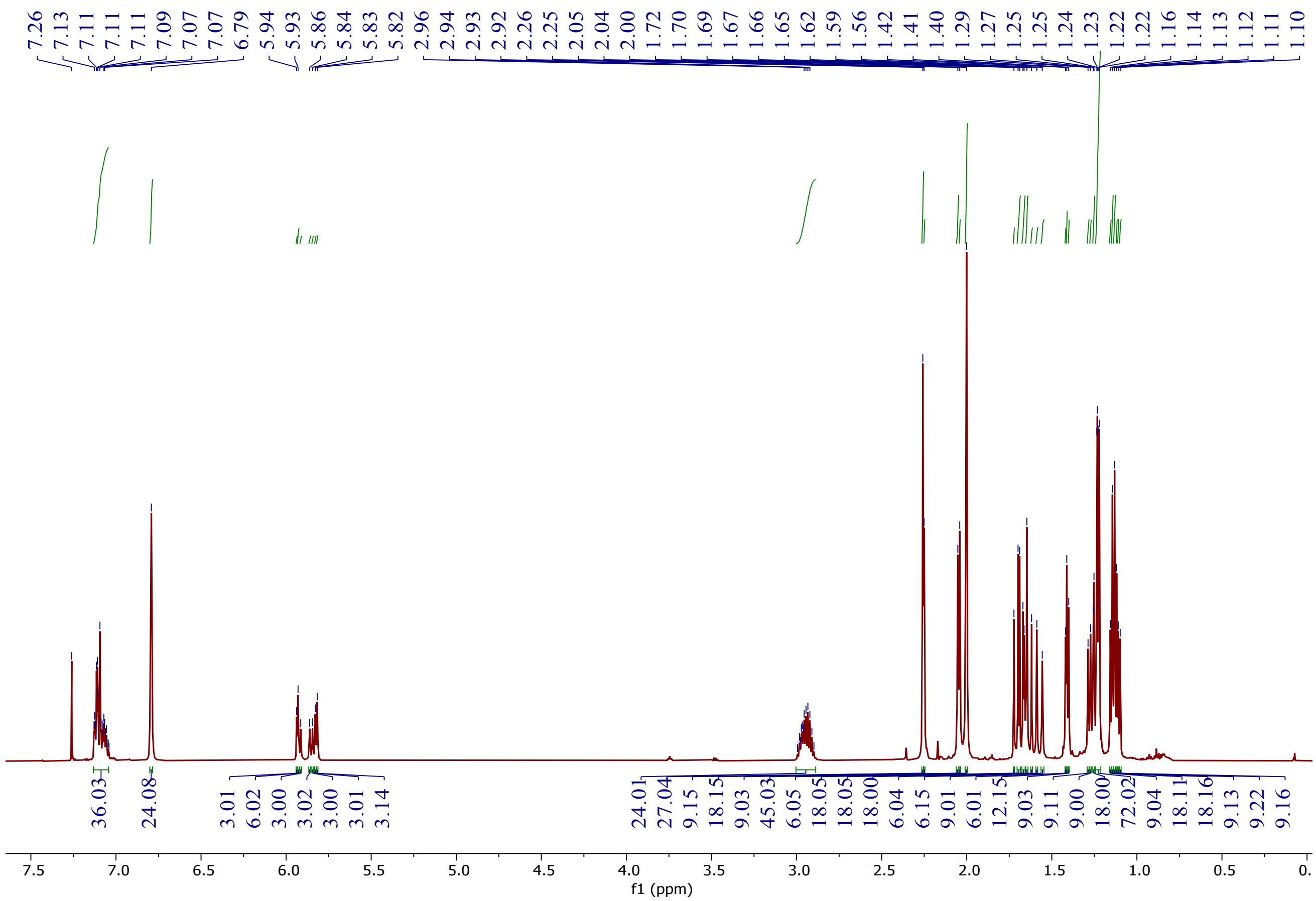

Figure S1 ^{1}H NMR of the **SP1** mixture composed of $[\mathbf{L_A}Cu]_3$ and $[\mathbf{L_B}Cu]_3$ in 1:1 molar ratio recorded in $CDCl_3$

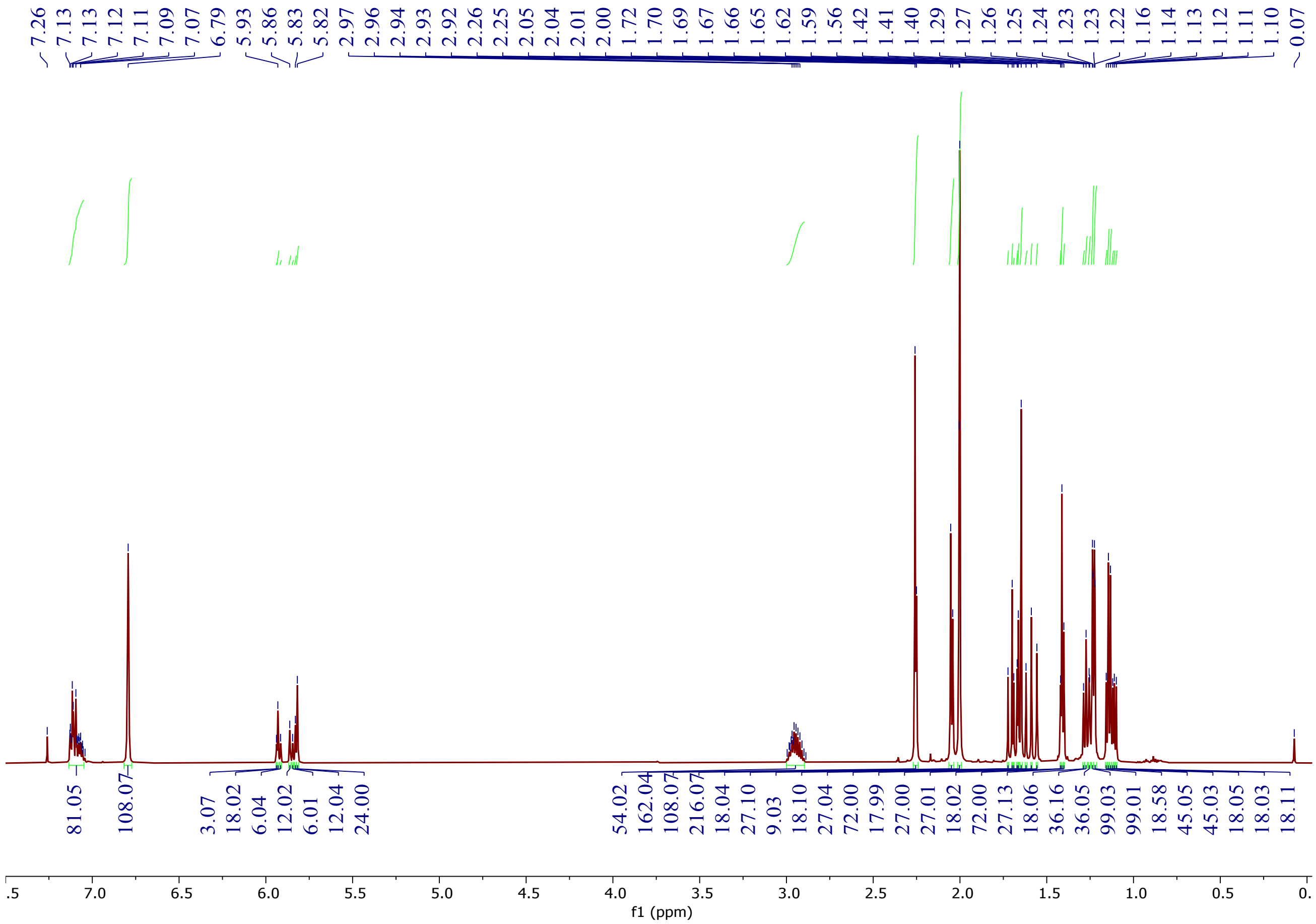

Figure S2 ¹H NMR of the **SP1** mixture composed of [$\mathbf{L_A}$Cu]$_3$ and [$\mathbf{L_B}$Cu]$_3$ in 2:1 molar ratio recorded in $CDCl_3$

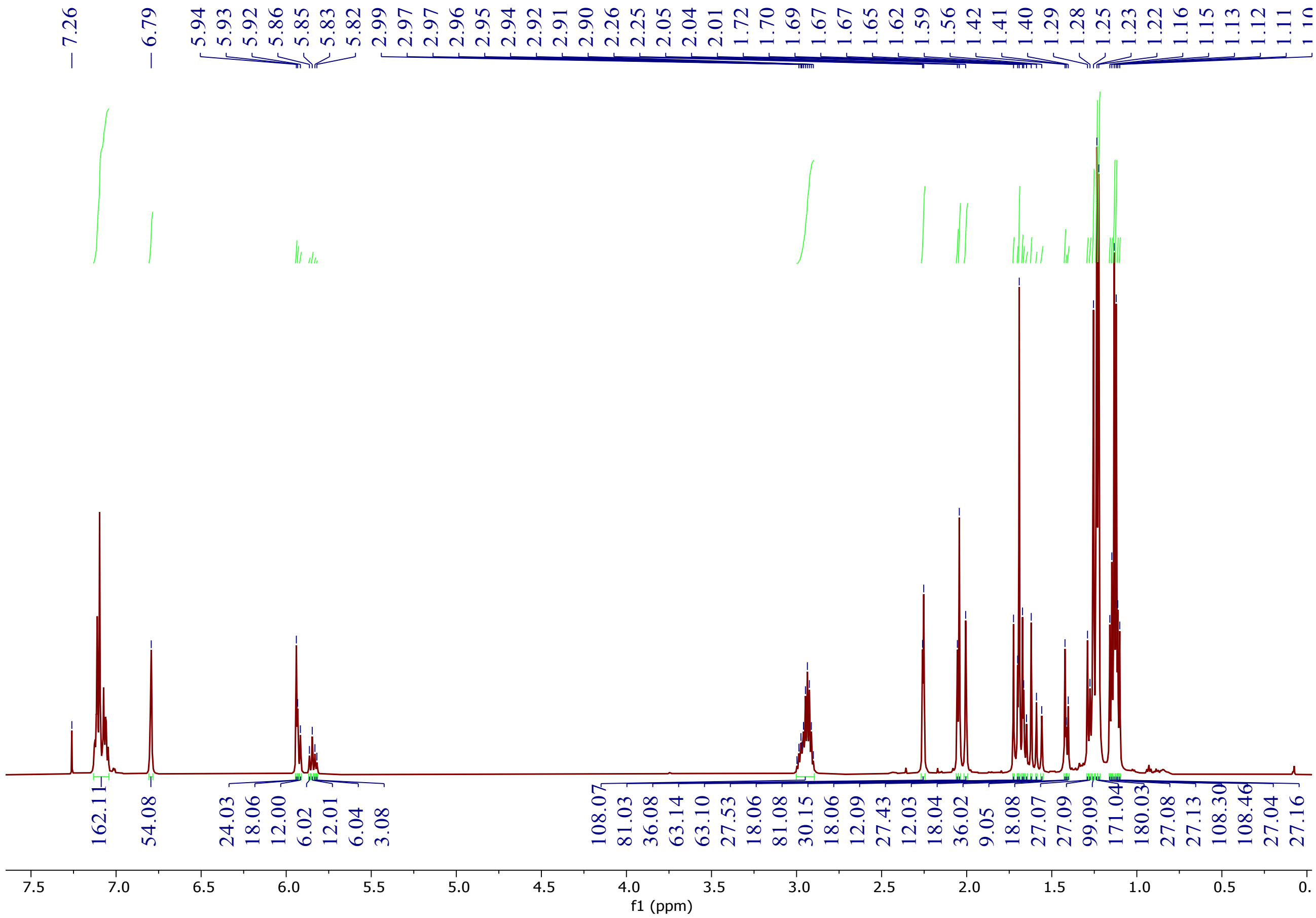

Figure S3 ^{1}H NMR of the **SP1** mixture composed of [$\mathbf{L_A}$Cu]$_3$ and [$\mathbf{L_B}$Cu]$_3$ in 1:2 molar ratio recorded in $CDCl_3$

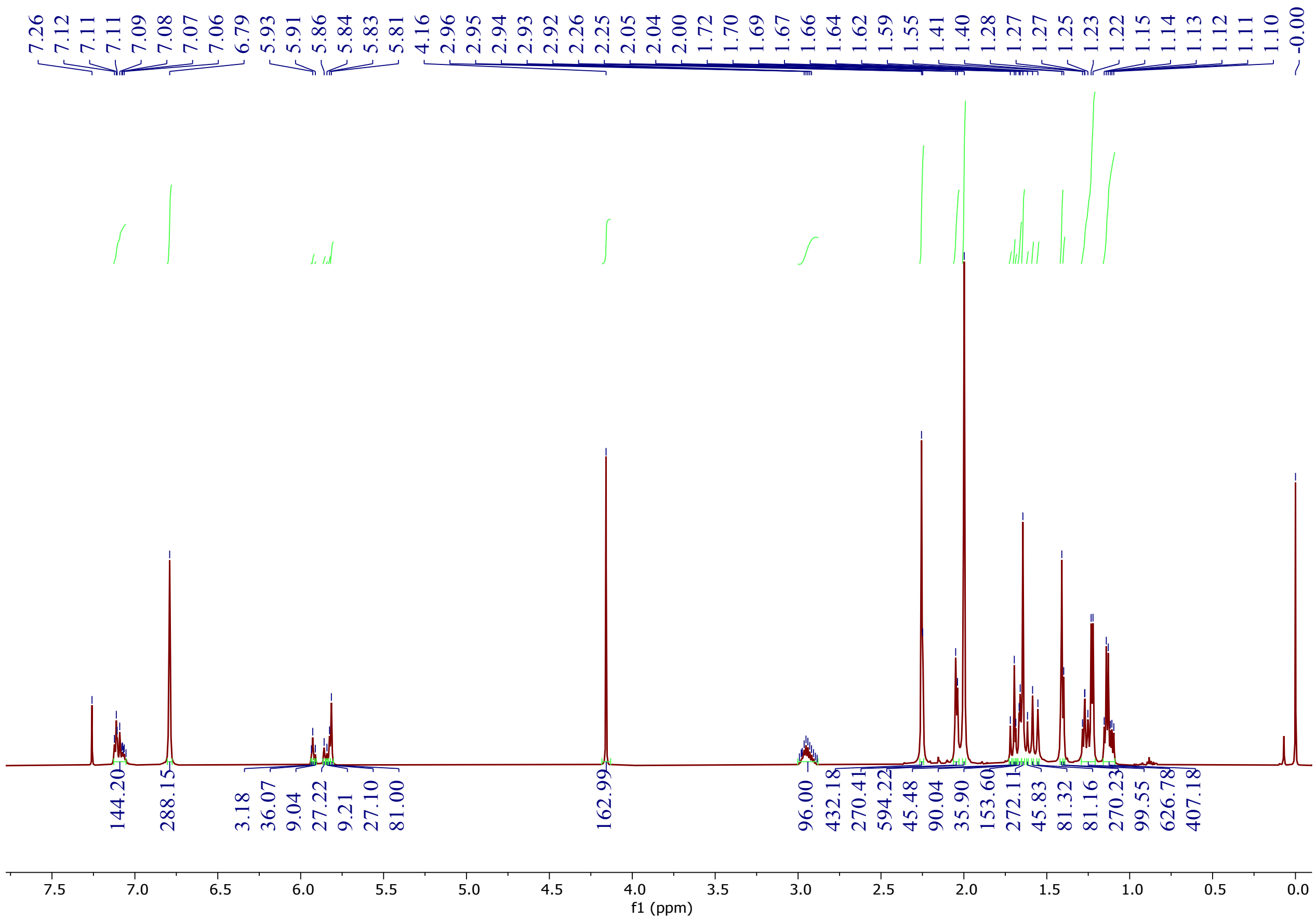

Figure S4 ^{1}H NMR of the **SP1** mixture composed of [$\mathbf{L_A}$Cu]$_3$ and [$\mathbf{L_B}$Cu]$_3$ in 3:1 molar ratio recorded in $CDCl_3$

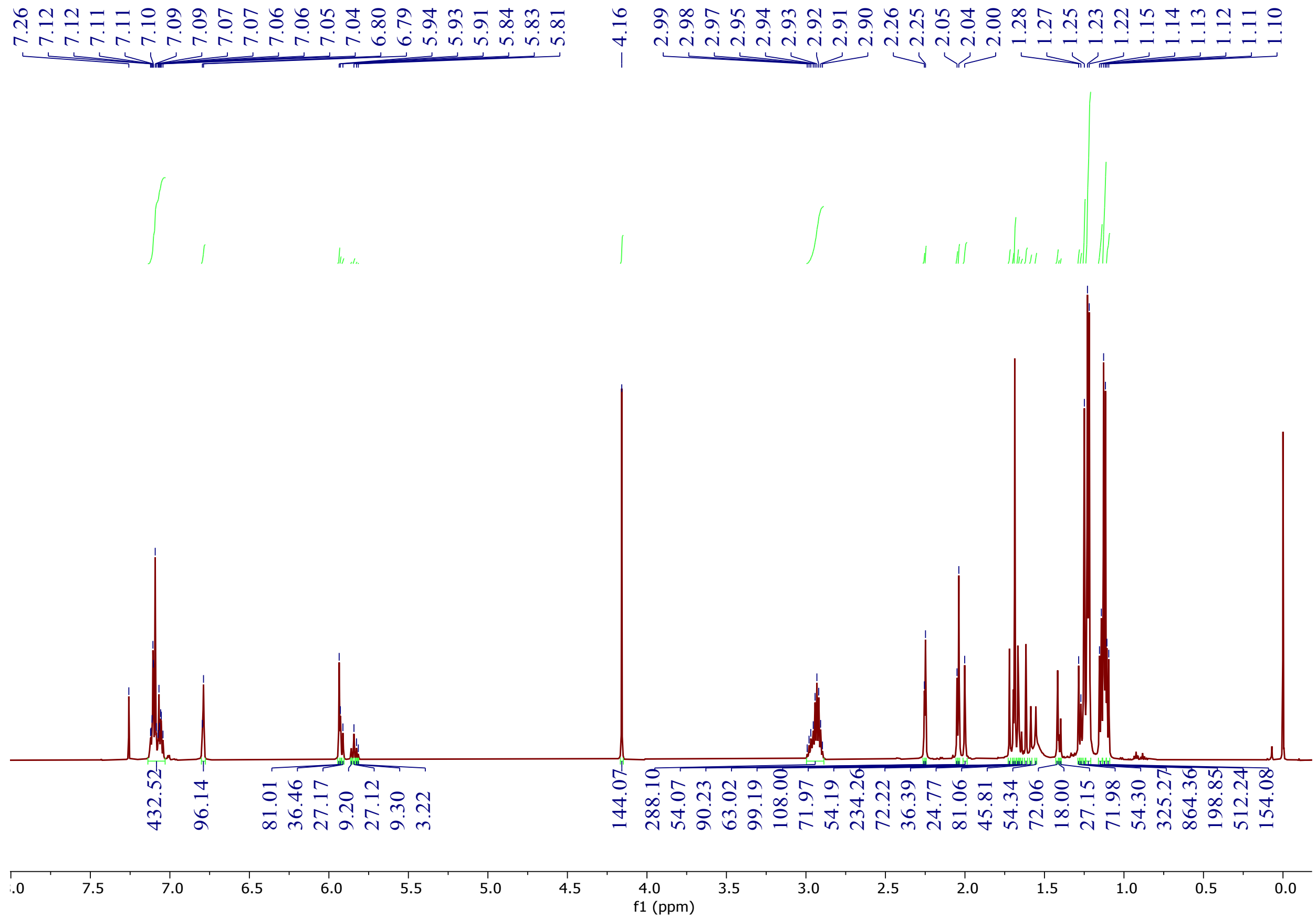

Figure S5 ^{1}H NMR of the **SP1** mixture composed of [$\mathbf{L_A}$Cu]$_3$ and [$\mathbf{L_B}$Cu]$_3$ in 1:3 molar ratio recorded in $CDCl_3$

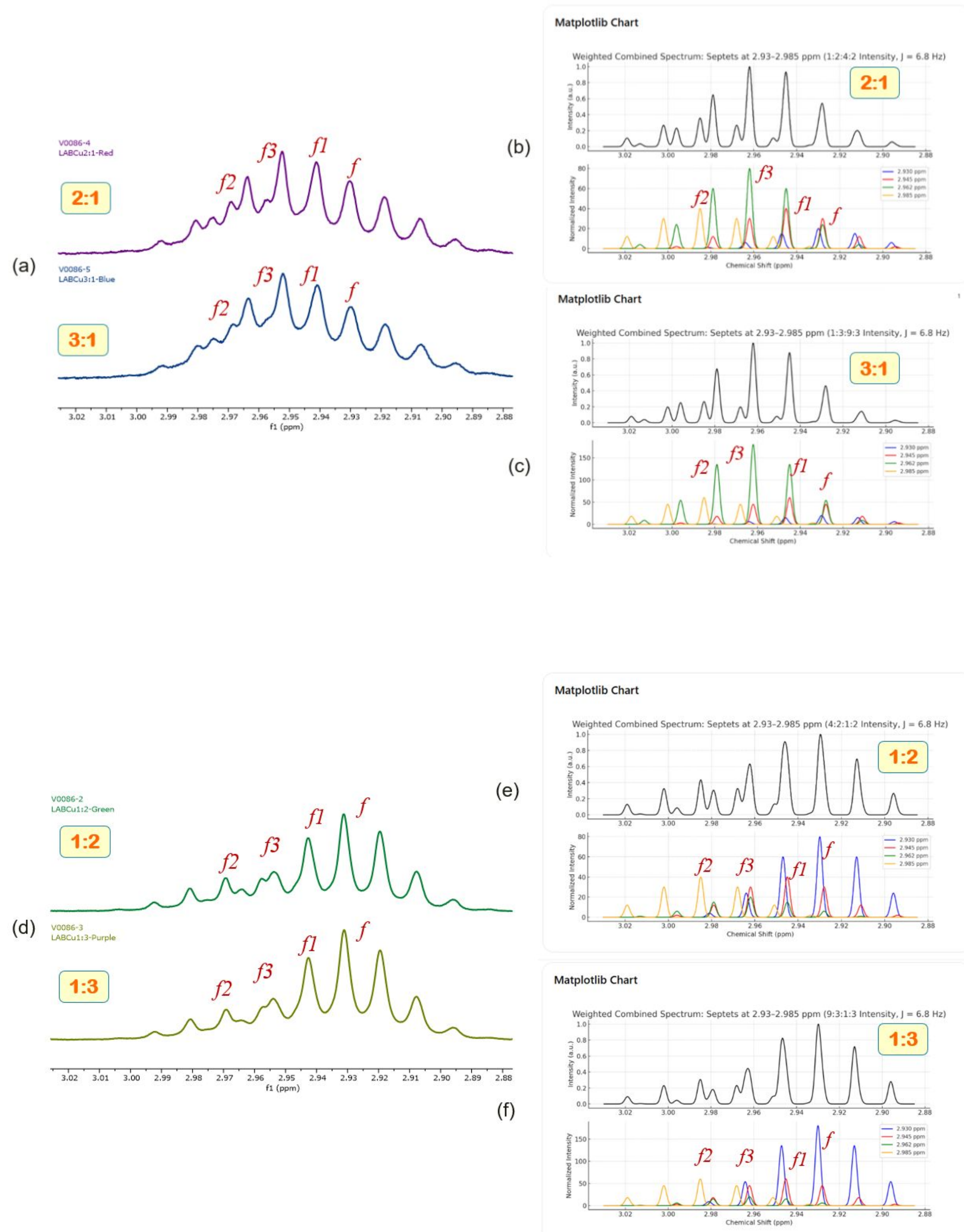

Figure S6. (a) Illustration of experimental results of 600 MHz ^{1}H NMR simulations of **SP1** mixture corresponding to ^{i}pr-C*H* protons in 2:1, and 3:1, ratios. (d) experimental simulations for 1:2 and 1:3 ratios. (b) AI-illustrations of ^{1}H NMR results of **SP1** mixture corresponding to ^{i}pr-C*H* protons corresponding to the 2:1, and (c) 3:1 ratio, (e) 1:2, and (f) 1:3 ratios.

Variable Temperature ^{1}H NMR Results

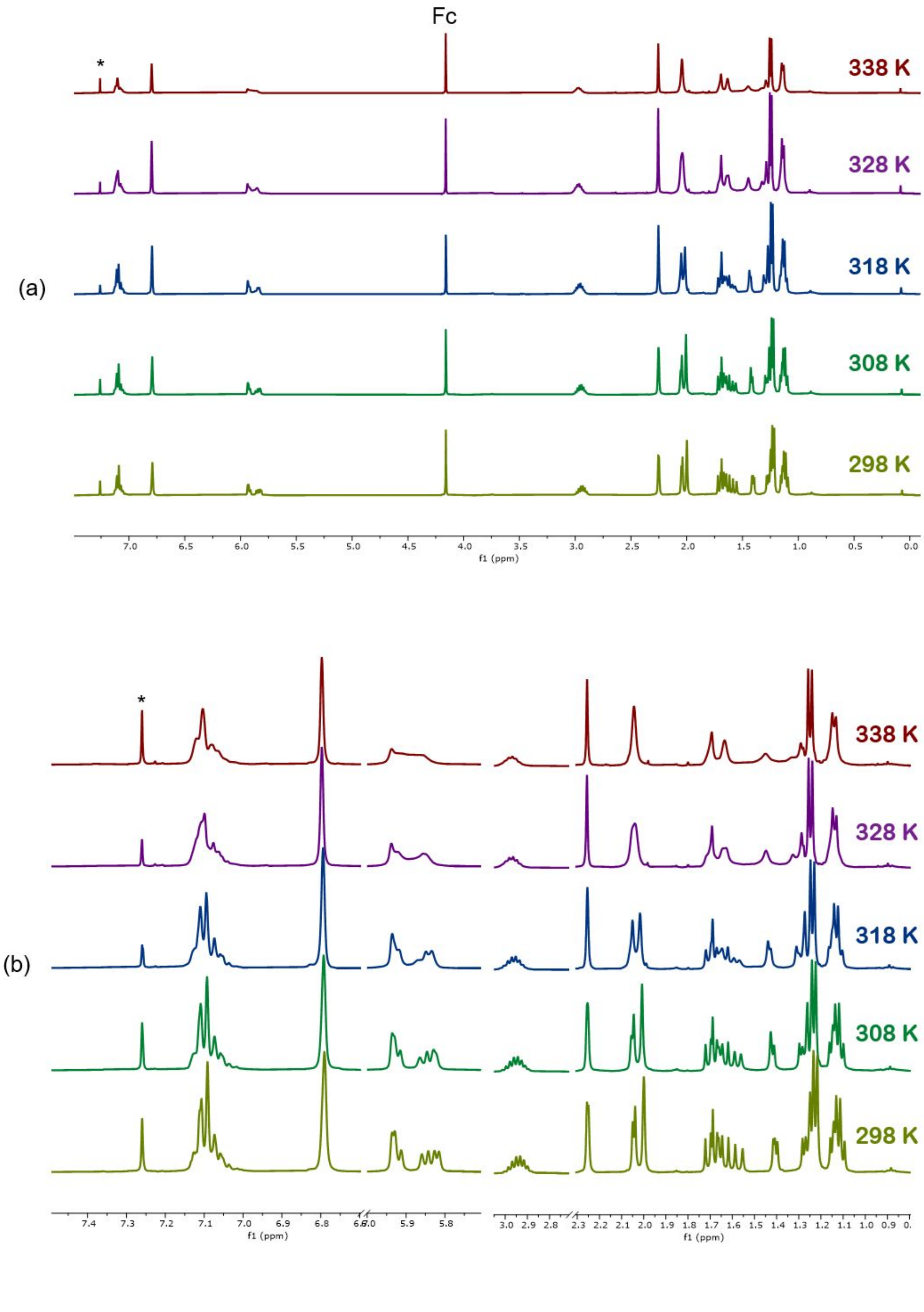

Figure S7. (a) Variable-temperature ^{1}H NMR spectra of the **SP1** (1:1) mixture in $CDCl_3$ recorded from 298 to 338 K, showing temperature-dependent spectral evolution associated with dynamic exchange within the A3/B3 combinatorial library. Ferrocene (Fc) was used as an internal standard, and the signal marked with (*) corresponds to residual solvent. (b) Expanded view of selected spectral regions highlighting temperature-dependent chemical shift changes, signal broadening, and progressive resolution of resonances upon cooling.

(a)

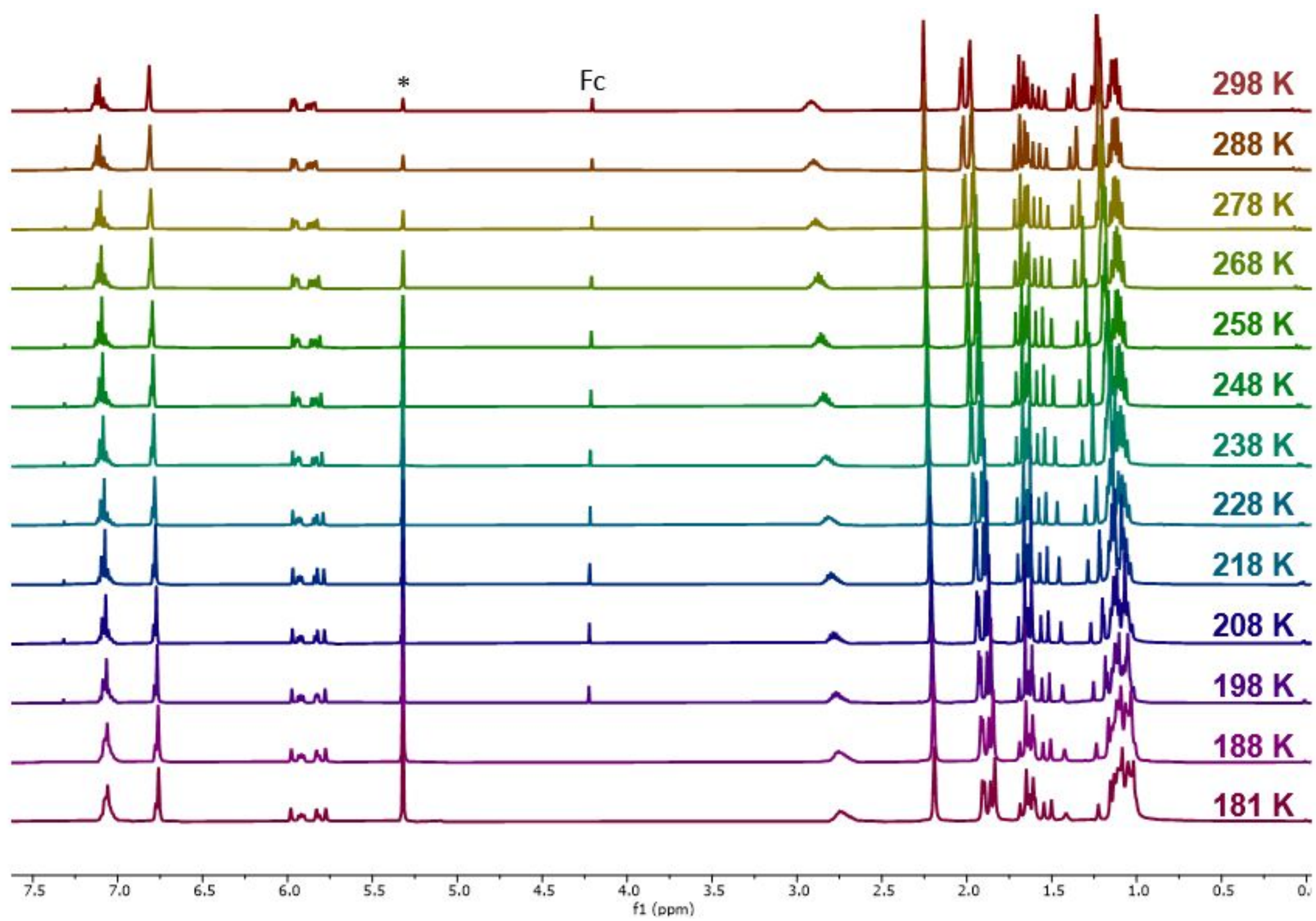

(b)

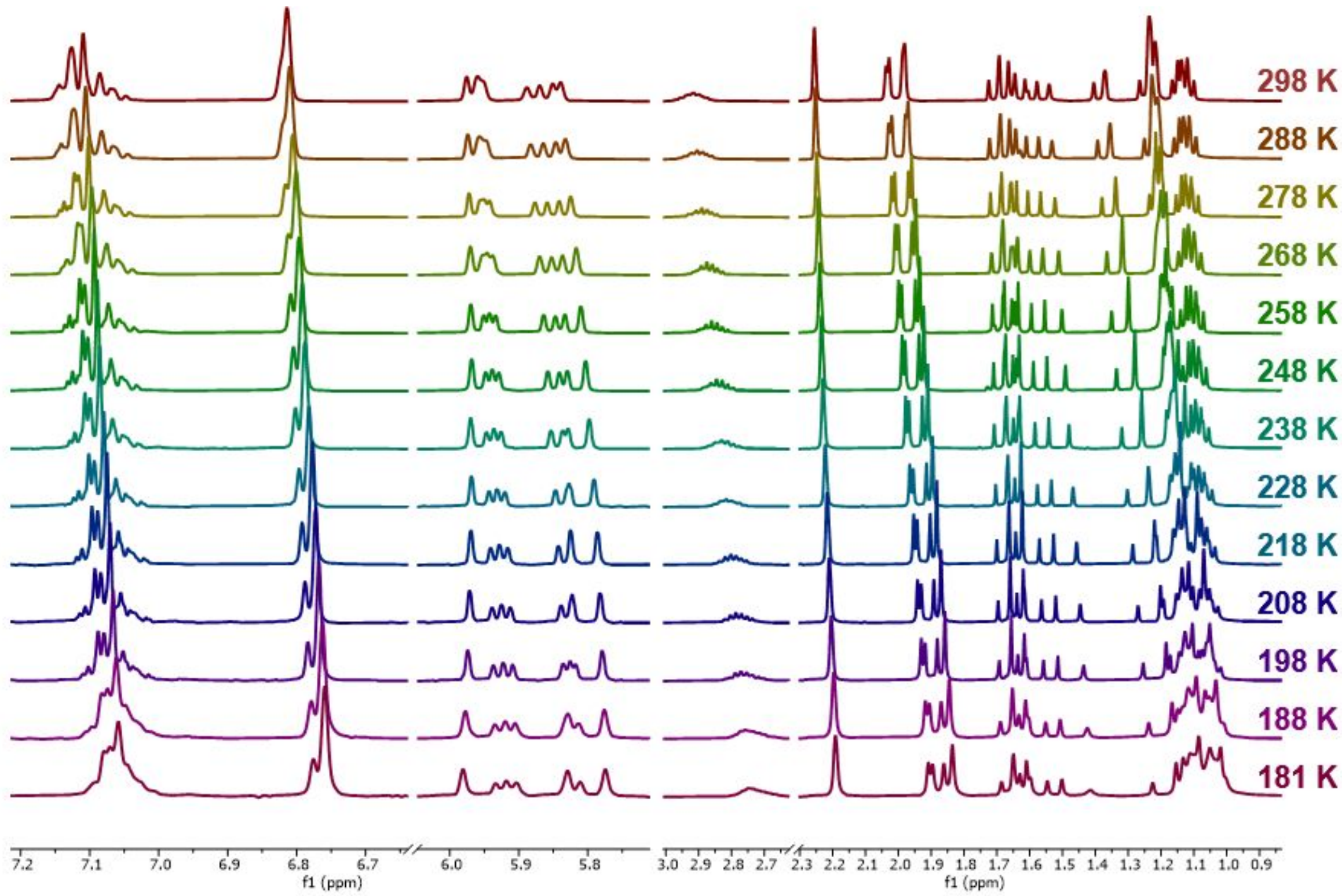

Figure S8. (a) Variable-temperature ¹H NMR spectra of the **SP1** (1:1) mixture in CD_2Cl_2 recorded from 298 to 181 K, showing temperature-dependent spectral evolution associated with dynamic exchange within the **A3/B3** combinatorial library. Ferrocene (Fc) was used as an internal standard, and the signal marked with (*) corresponds to residual solvent. (b) Expanded view of selected spectral regions highlighting temperature-dependent chemical shift changes, signal broadening, and progressive resolution of resonances upon cooling.

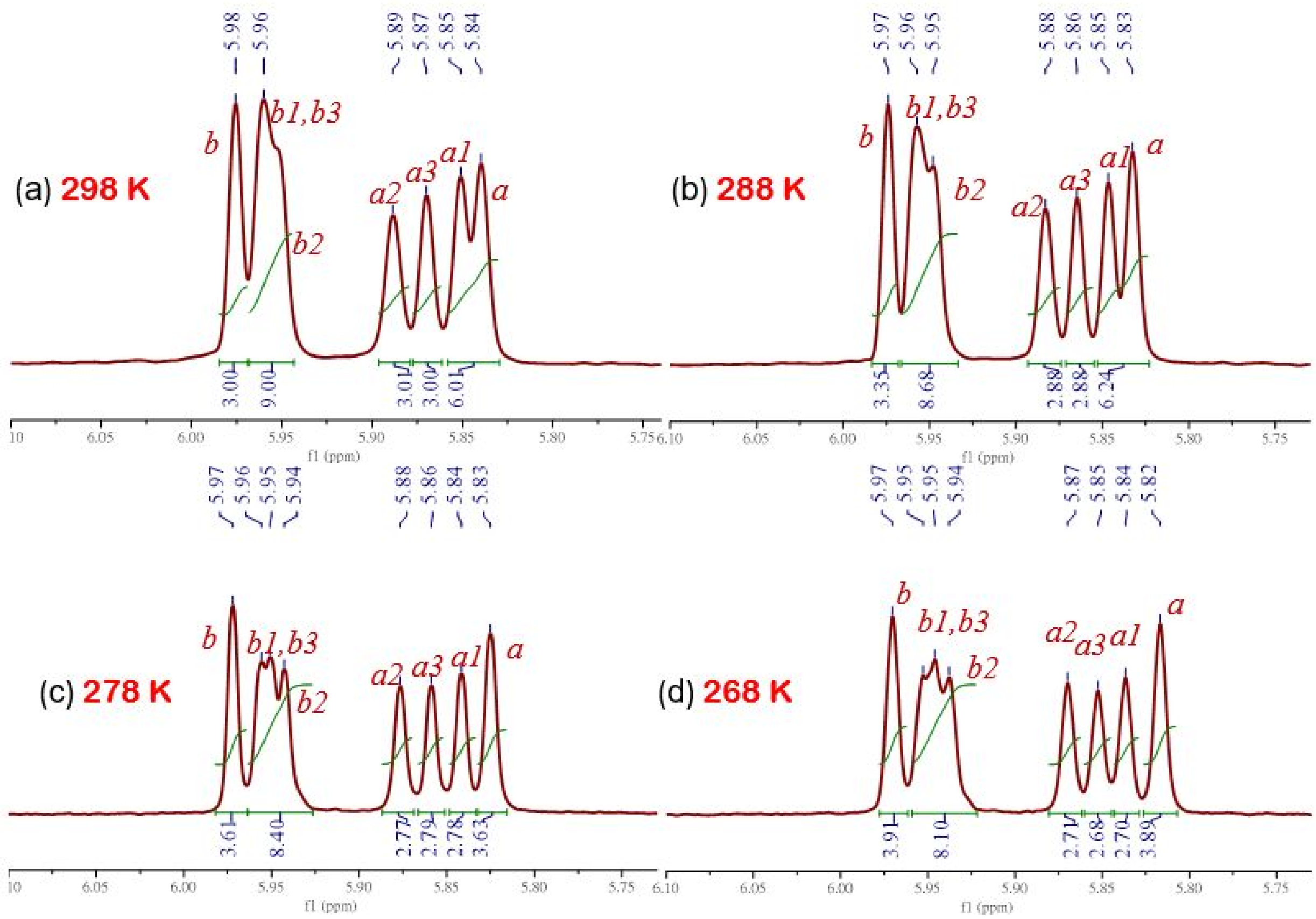
(a) 298 K
(b) 288 K
(c) 278 K
(d) 268 K
b
b1,b3
b2
a2
a3
a1
a
f1 (ppm)

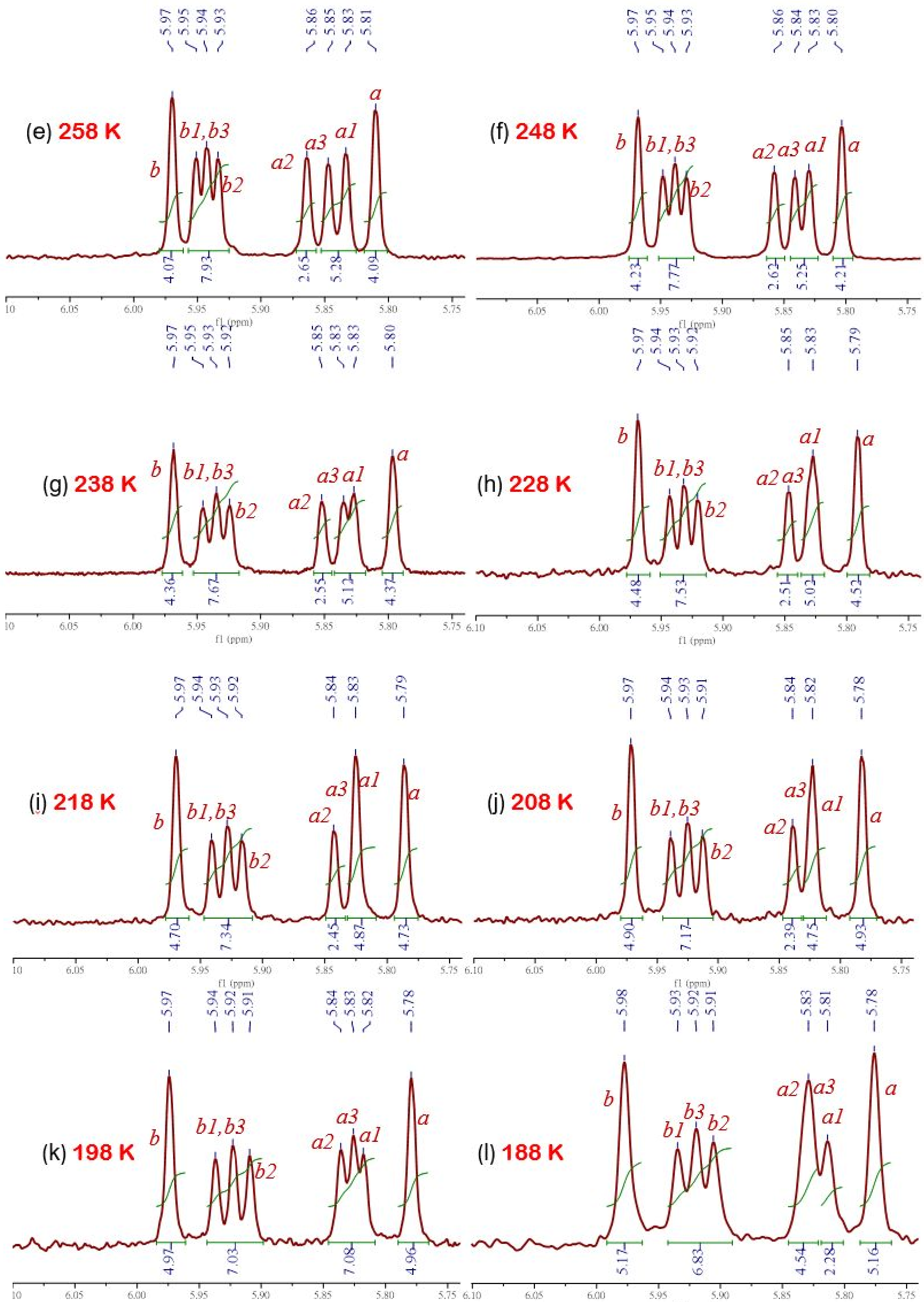

(e) 258 K
5.97 5.95 5.94 5.93 5.86 5.85 5.83 5.81
b b1,b3 b2 a2 a3 a1 a
4.07 7.93 2.65 5.28 4.09
(f) 248 K
5.97 5.95 5.94 5.93 5.86 5.84 5.83 5.80
b b1,b3 b2 a2 a3 a1 a
4.23 7.77 2.62 5.25 4.21
(g) 238 K
5.97 5.95 5.93 5.92 5.85 5.83 5.83 5.80
b b1,b3 b2 a2 a3 a1 a
4.36 7.67 2.55 5.12 4.37
(h) 228 K
5.97 5.94 5.93 5.92 5.85 5.83 5.79
b b1,b3 b2 a2 a3 a1 a
4.48 7.53 2.51 5.02 4.52
(i) 218 K
5.97 5.94 5.93 5.92 5.84 5.83 5.79
b b1,b3 b2 a2 a3 a1 a
4.70 7.34 2.45 4.87 4.73
(j) 208 K
5.97 5.94 5.93 5.91 5.84 5.82 5.78
b b1,b3 b2 a2 a3 a1 a
4.90 7.17 2.39 4.75 4.93
(k) 198 K
5.97 5.94 5.92 5.91 5.84 5.83 5.82 5.78
b b1,b3 b2 a2 a3 a1 a
4.97 7.03 7.08 4.96
(l) 188 K
5.98 5.93 5.92 5.91 5.83 5.81 5.78
b b1 b3 b2 a2 a3 a1 a
5.17 6.83 4.54 2.28 5.16
f1 (ppm)

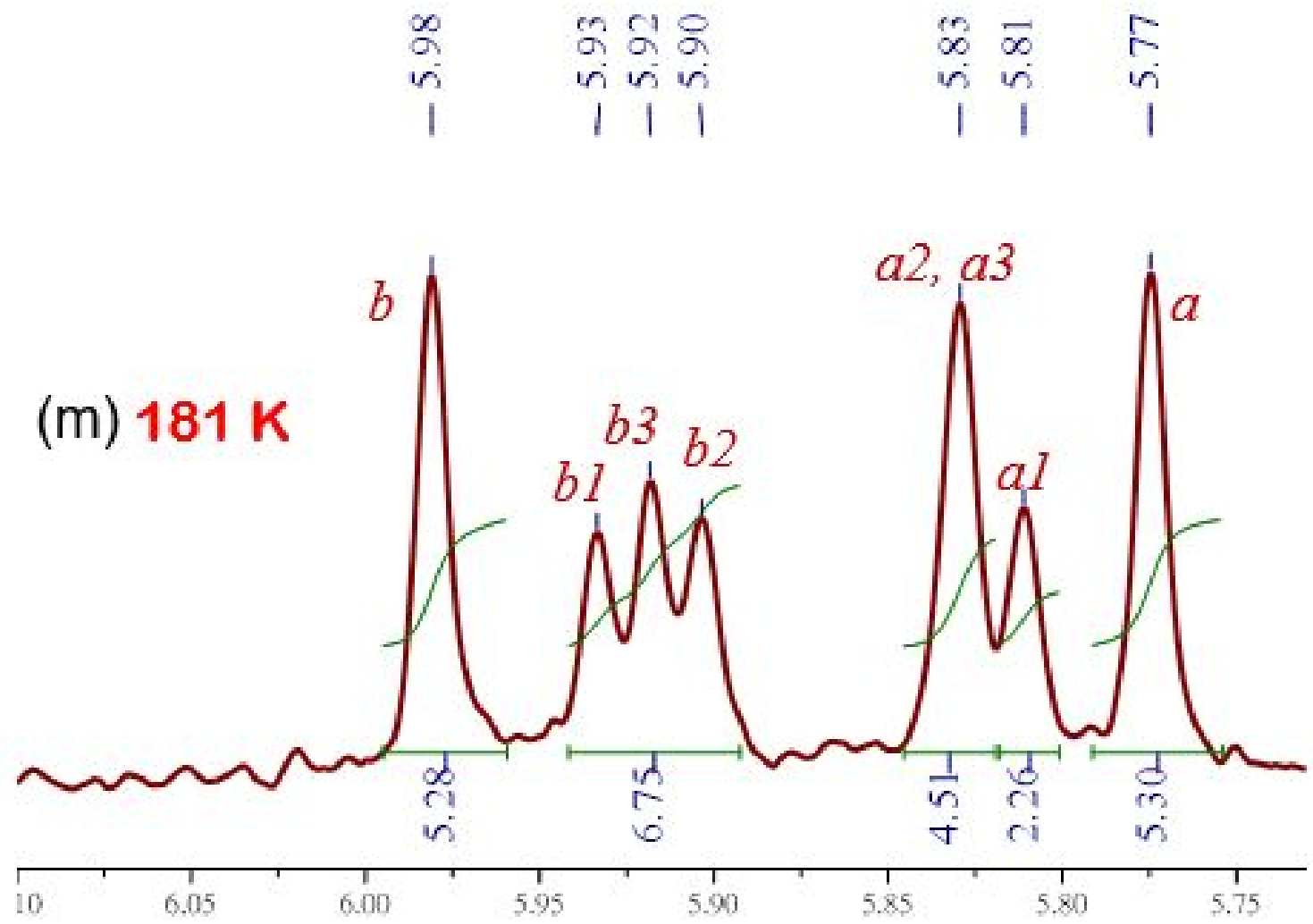

Figure S9. Expanded view of the methine (–CH) backbone region (δ = 5.74–6.00 ppm) of **SP1** in CD_2Cl_2, showing temperature-dependent chemical shift changes from 298 K to 181 K. Spectra recorded at (a) 298 K, (b) 288 K, (c) 278 K, (d) 268 K, (e) 258 K, (f) 248 K, (g) 238 K, (h) 228 K, (i) 218 K, (j) 208 K, (k) 198 K, (l) 188 K, and (m) 181 K.

Table S1. Temperature-dependent ^{1}H NMR integration values of methine (–CH) proton resonances assigned to A- and B-derived coordination units (a, a1–a3 and b, b1–b3) for the **A3/B3** dynamic combinatorial system in CD_2Cl_2.

T (K)	***a1***	***a2***	***b3***	***b1***	***b2***	***a3***	***a***	***b***
298	3.00	3.00	3.00	3.00	3.00	3.00	3.00	3.00
288	2.89	2.89	2.88	2.88	2.88	2.89	3.35	3.35
278	2.78	2.77	2.77	2.77	2.77	2.79	3.61	3.63
268	2.70	2.71	2.73	2.73	2.73	2.68	3.91	3.89
258	2.64	2.65	2.64	2.64	2.64	2.64	4.07	4.09
248	2.59	2.62	2.61	2.61	2.61	2.62	4.23	4.21
238	2.56	2.55	2.56	2.56	2.56	2.56	4.36	4.37
228	2.51	2.51	2.51	2.51	2.51	2.51	4.48	4.52
218	2.43	2.45	2.44	2.44	2.44	2.43	4.70	4.73
208	2.37	2.39	2.36	2.36	2.36	2.37	4.93	4.90
198	2.36	2.36	2.34	2.34	2.34	2.36	4.96	4.97
188	2.28	2.27	2.27	2.27	2.27	2.27	5.07	5.07
181	2.26	2.25	2.25	2.25	2.25	2.25	5.30	5.28

Table S2. Calculation of equilibrium constants *K* for 1:1 molar ratio A3B3 self-assembly by Variable Temperature ^{1}H NMR analysis from Figure S9

$$\mathrm{K} = \frac{[\mathrm{A2B}][\mathrm{AB2}]}{[\mathrm{A3}][\mathrm{B3}]} = \frac{[a1+a2+b3][b1+b2+a3]}{[\mathrm{a}][\mathrm{b}]}$$

$$\mathrm{K}_{298\ \mathrm{K}} = \frac{[3.00+3.00+3.00][3.00+3.00+3.00]}{[3.00][3.00]} = \frac{81.00}{9.00} = 9.00$$

$$\mathrm{K}_{288\ \mathrm{K}} = \frac{[2.89+2.89+2.88][2.88+2.88+2.89]}{[3.35][3.35]} = \frac{74.91}{11.22} = 6.67$$

$$\mathrm{K}_{278\ \mathrm{K}} = \frac{[2.78+2.77+2.77][2.77+2.77+2.79]}{[3.61][3.63]} = \frac{69.30}{13.10} = 5.29$$

$$\mathrm{K}_{268\ \mathrm{K}} = \frac{[2.70+2.71+2.73][2.73+2.73+2.68]}{[3.91][3.89]} = \frac{66.26}{15.20} = 4.35$$

$$\mathrm{K}_{258\ \mathrm{K}} = \frac{[2.64+2.65+2.64][2.64+2.64+2.64]}{[4.07][4.09]} = \frac{62.80}{16.64} = 3.77$$

$$\mathrm{K}_{248\ \mathrm{K}} = \frac{[2.59+2.62+2.61][2.61+2.61+2.62]}{[4.23][4.21]} = \frac{61.30}{17.80} = 3.44$$

$$\mathrm{K}_{238\ \mathrm{K}} = \frac{[2.56+2.55+2.56][2.56+2.56+2.56]}{[4.36][4.37]} = \frac{58.90}{19.05} = 3.09$$

$$\mathrm{K}_{228\ \mathrm{K}} = \frac{[2.51+2.51+2.51][2.51+2.51+2.51]}{[4.48][4.52]} = \frac{56.70}{20.25} = 2.80$$

$$\mathrm{K}_{218\ \mathrm{K}} = \frac{[2.43+2.45+2.44][2.44+2.44+2.43]}{[4.70][4.73]} = \frac{53.40}{22.23} = 2.40$$

$$\mathrm{K}_{208\ \mathrm{K}} = \frac{[2.37+2.39+2.36][2.36+2.36+2.37]}{[4.93][4.90]} = \frac{50.48}{24.16} = 2.09$$

$$\mathrm{K}_{198\ \mathrm{K}} = \frac{[2.36+2.36+2.34][2.34+2.34+2.36]}{[4.96][4.97]} = \frac{47.78}{24.65} = 1.94$$

$$\mathrm{K}_{188\ \mathrm{K}} = \frac{[2.28+2.27+2.27][2.27+2.27+2.27]}{[5.07][5.07]} = \frac{46.44}{25.70} = 1.81$$

$$\mathrm{K}_{181\ \mathrm{K}} = \frac{[2.26+2.25+2.25][2.25+2.25+2.25]}{[5.30][5.28]} = \frac{45.63}{27.98} = 1.63$$

Equilibrium constants were determined from VT ^{1}H NMR integrations of methine (–C*H*) resonances corresponding to homoleptic and heteroleptic tricopper clusters. Signals *a* and *b* were assigned to **A3** and **B3**, respectively, while grouped integrations **A2B** (*a1+a2+b3)* and **AB2** (*b1+b2+a3*) represent contributions from heteroleptic assemblies. The equilibrium analysis assumes reversible redistribution according to **A3+B3 ⇌ A2B+AB2**. Ferrocene (Fc) was added as an internal standard relative to one ligand–copper coordination unit, and its integration value was

fixed at $\frac{10\,H}{3}$ = 3.33 for normalization of all temperature-dependent spectra. The resulting temperature-dependent *K* values support a dynamic, statistically governed self-assembly process driven by Cu–ligand exchange.

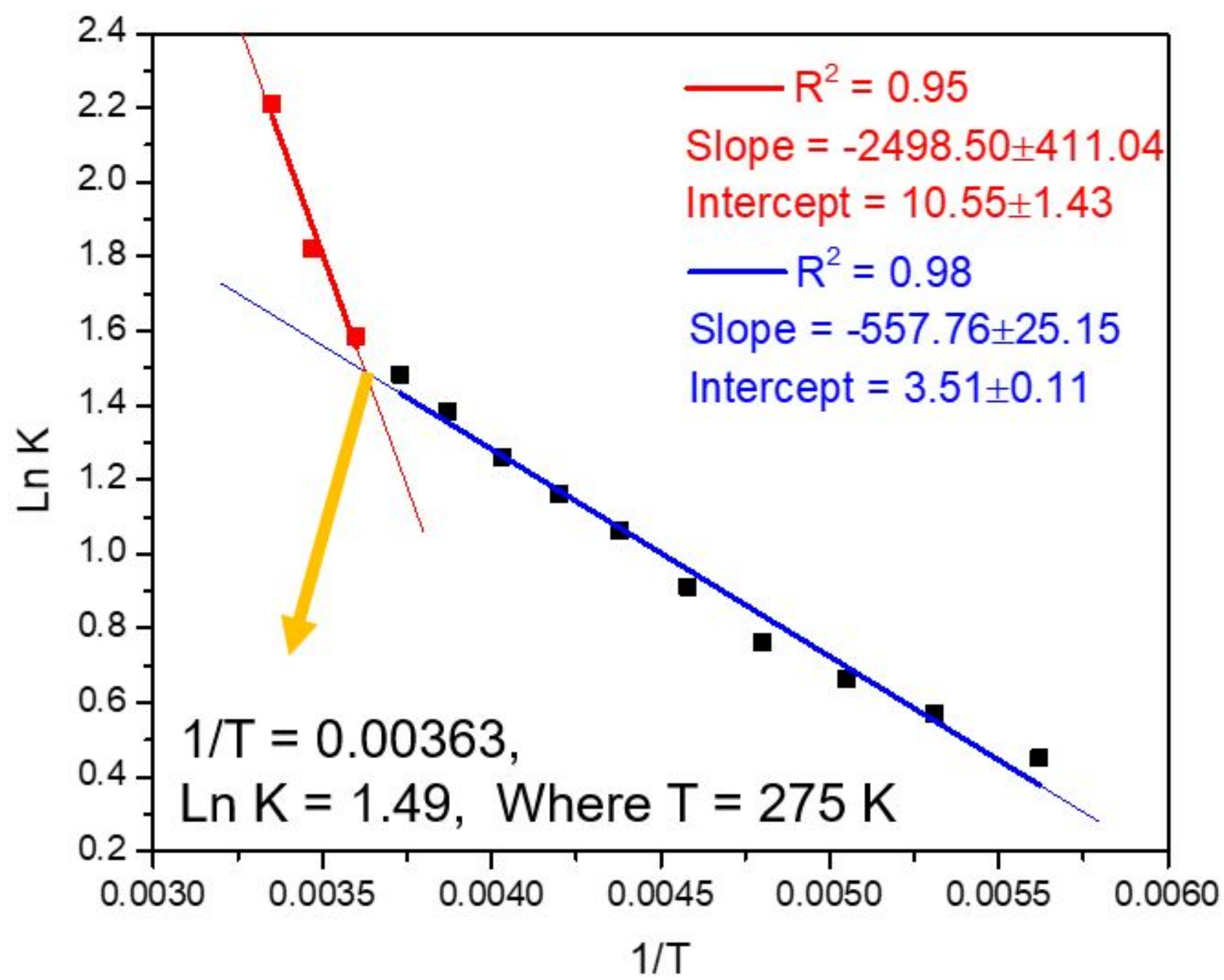

Calculations of ΔH and ΔS based on Figure 5b

Red Plot

Slope (m) = $(-\frac{\Delta H}{R})$ = -2498.5±411.04

⇒ ΔH = 2498.5±411 x 8.314

⇒ ΔH = 20772.53±3417 J/mol

⇒ **ΔH = 20.772±3.417 kJ/mol**

Intercept (C) = $(\frac{\Delta S}{R})$ = 10.55±1.43

⇒ ΔS = 10.55±1.43 x 8.314

Blue Plot

Slope (m) = $(-\frac{\Delta H}{R})$ = -557.76±25.15

⇒ ΔH = 557.76 ±25.15x 8.314

⇒ ΔH = 4637.02±209.1 J/mol

⇒ **ΔH = 4.637±0.209 kJ/mol**

Intercept (C) = $(\frac{\Delta S}{R})$ = 3.51±0.11

⇒ ΔS = 3.51±0.11 x 8.314

$\Rightarrow$ $\Delta S = 87.713 \pm 11.8$ J/mol K

$\Rightarrow$ **$\Delta S = 0.0877 \pm 0.012$ kJ/mol K**

$\Rightarrow$ $\Delta S = 29.18 \pm 0.91$ J/mol K

$\Rightarrow$ **$\Delta S = 0.0291 \pm 0.00091$ kJ/mol K**

ESI-MS Spectra

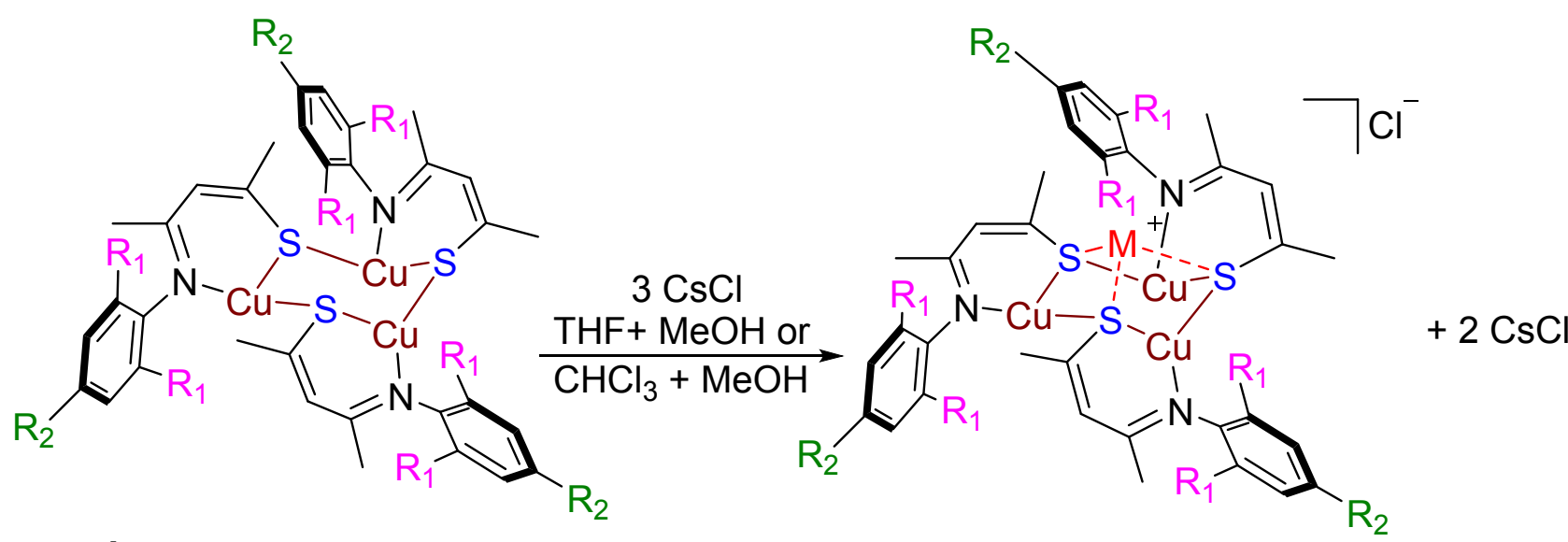

Scheme S1. Schematic representation of cesium coordinated tricopper clusters derived from *β*-thioketiminate tricopper clusters with CsCl.

These tricopper cluster adducts were obtained by cesium ions binding as soft metal salt, that shows a tendency to bind electrostatically with tricopper(I) clusters. The ESI-MS results for the cesium-bound tricopper(I) clusters reveals a minor fragment peak corresponding to the cesium-bound tricopper(I) cluster adduct full spectrum of ESI-MS is available below Figure S10. We were able to interpret and confirm the complex formations by their simulations, ensuring consistency with the corresponding ESI-MS fragment (see Figure S11-S13).

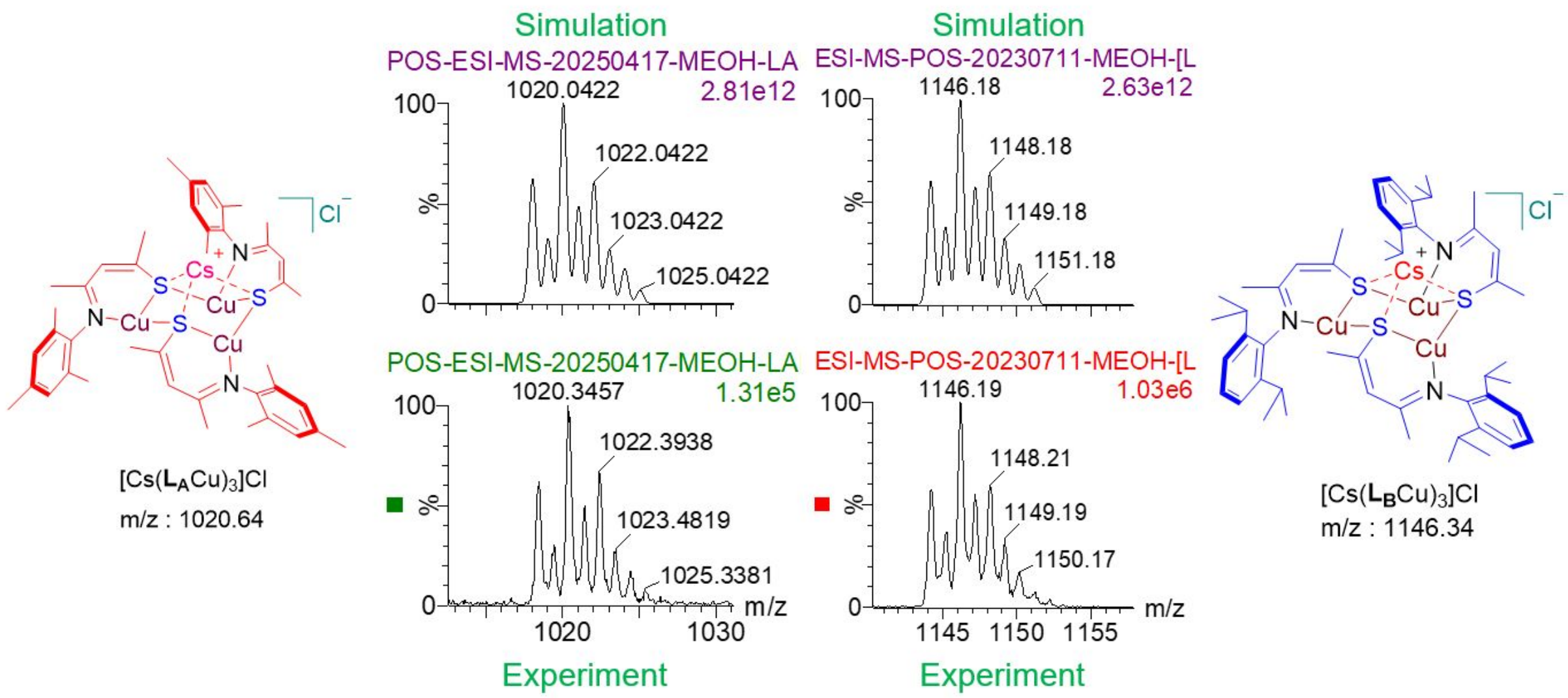

Figure S10. Interpretation of ESI-MS patterns corresponding to cesium-coordinated tricopper(I) clusters adducts.

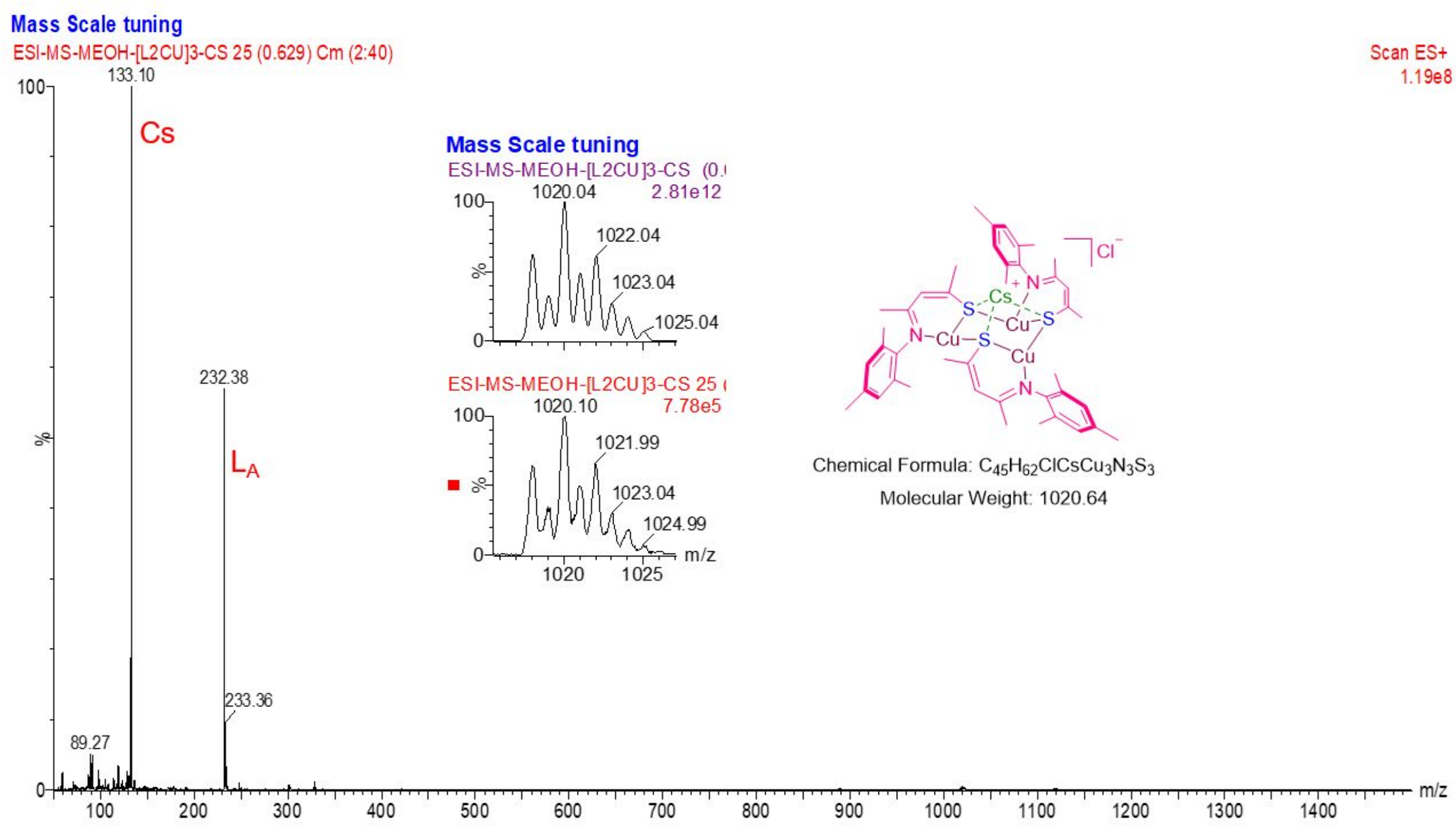

Figure S11. Full spectrum of ESI-MS peak correspond to tricopper(I) cluster **Cs@**[$\mathbf{L_A}$Cu]$_3$.

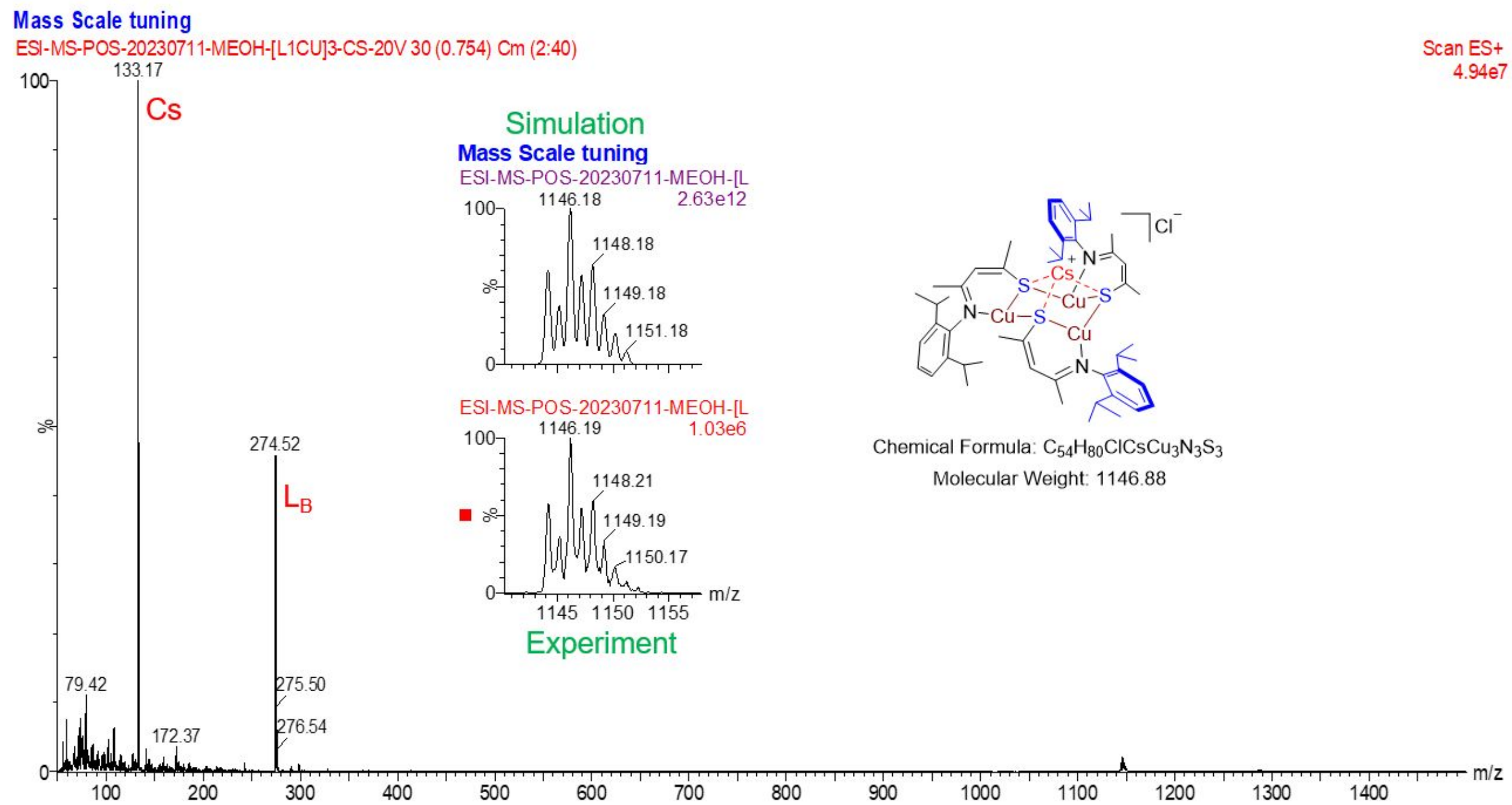

Figure S12. Full spectrum of ESI-MS peak correspond to tricopper(I) cluster **Cs@**[$\mathbf{L_B}$Cu]$_3$.

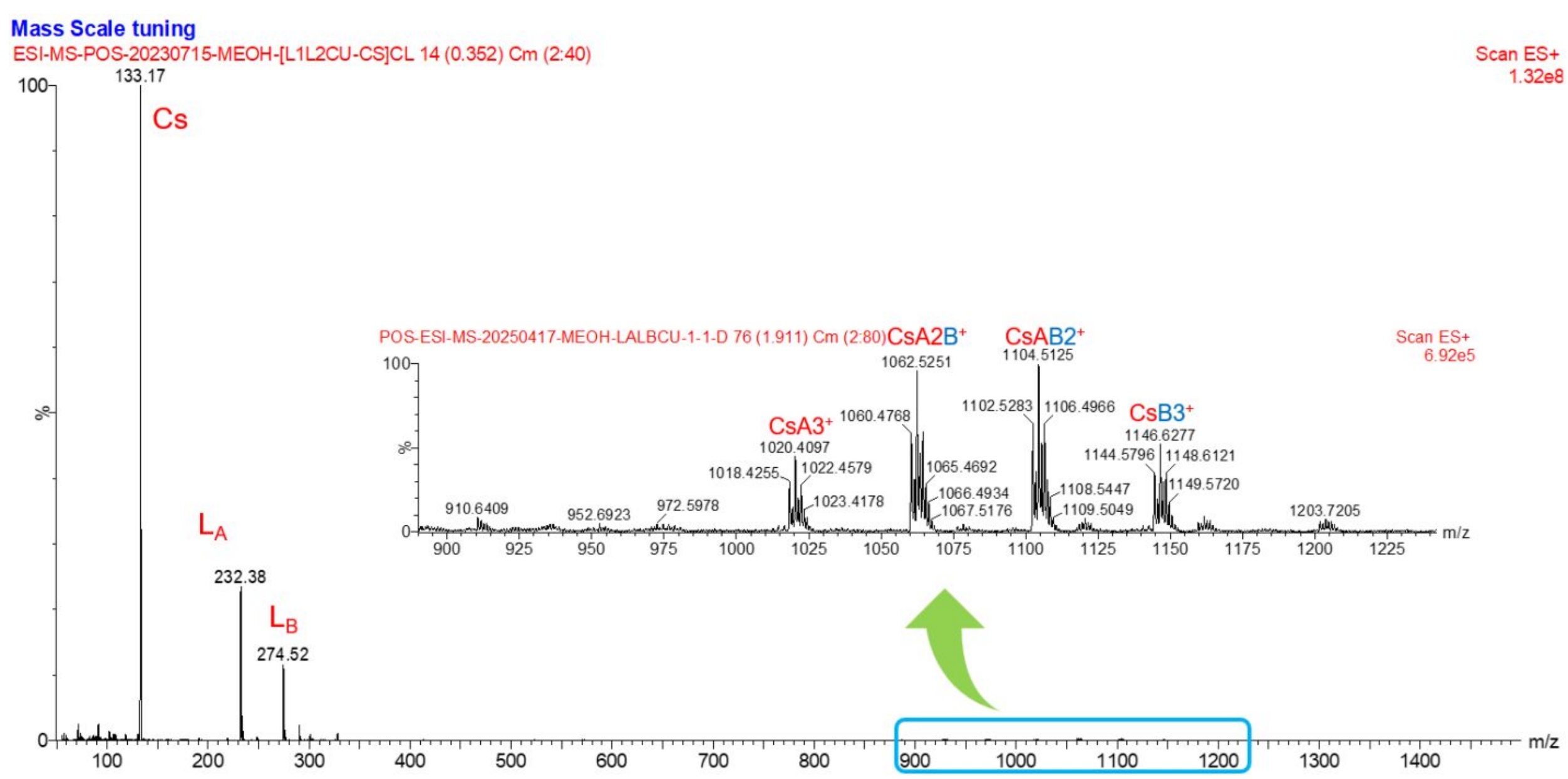

Figure S13. Full spectrum of ESI-MS spectra correspond to self-assembled product distribution 1:1 ratio of tricopper(I) clusters.

^{133}Cs NMR Titration Experimental Results

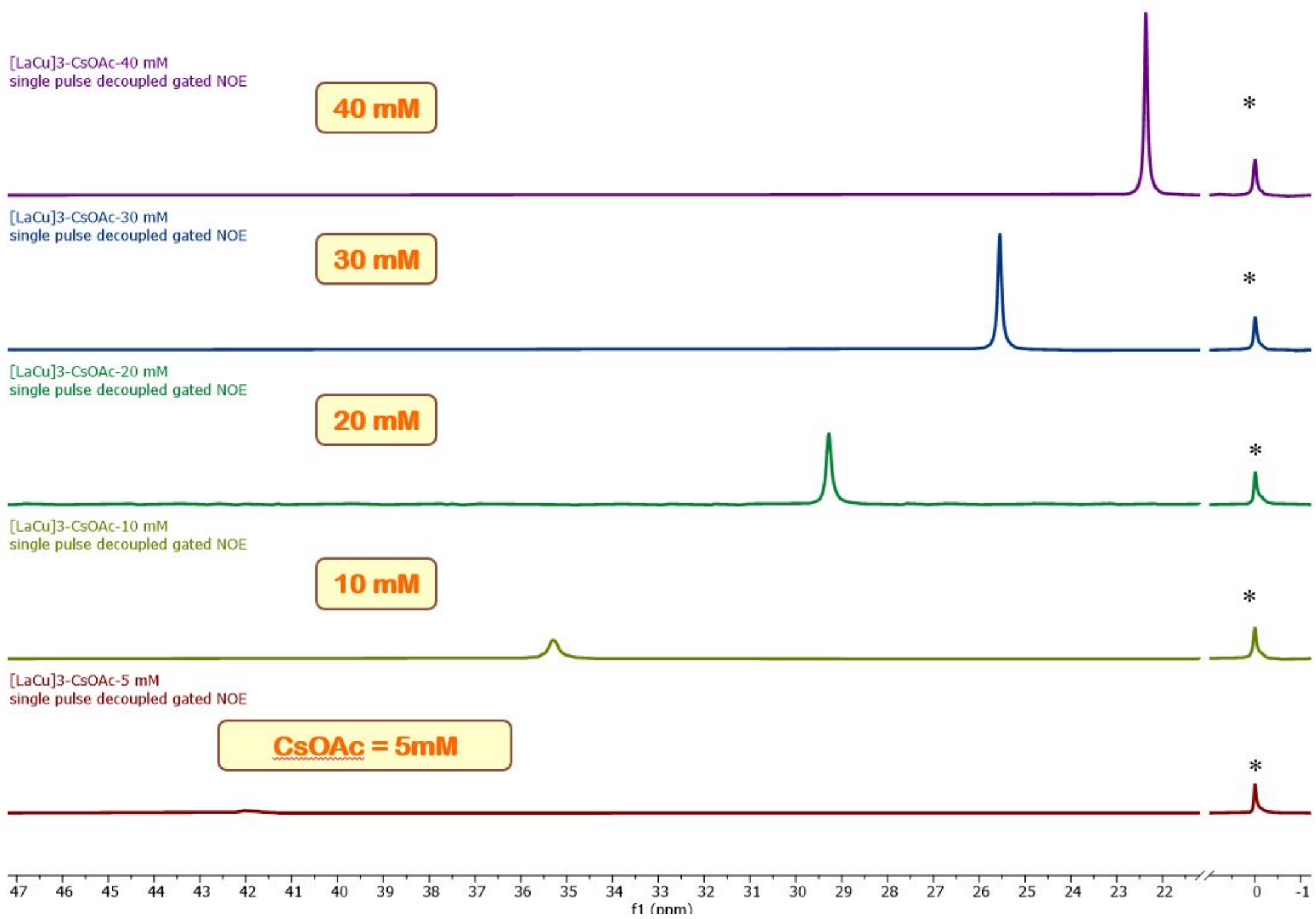

Figure S14. Illustration of ^{133}Cs NMR titration by treating incremental addition of CsOAc to [$\mathbf{L_A}$Cu]$_3$ to form **Cs@A3.** The resonance corresponding to pure CsOAc in MeOH, used as an internal standard, is marked with an asterisk (*)

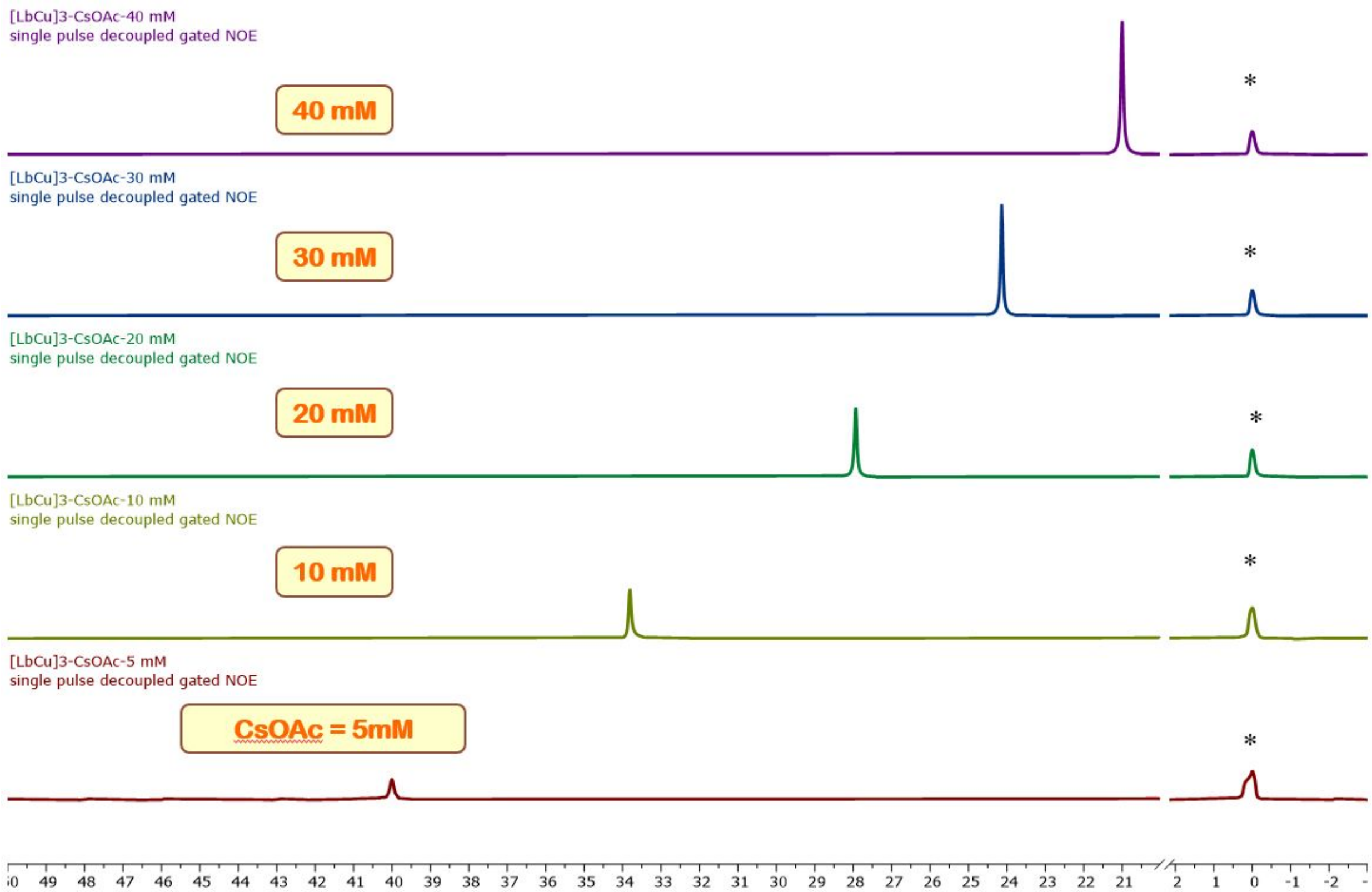

Figure S15. Illustration of ^{133}Cs NMR titration by treating incremental addition of CsOAc to [$\mathbf{L_B}$Cu]$_3$ to form **Cs@B3.** The resonance corresponding to pure CsOAc in MeOH, used as an internal standard, is marked with an asterisk (*)

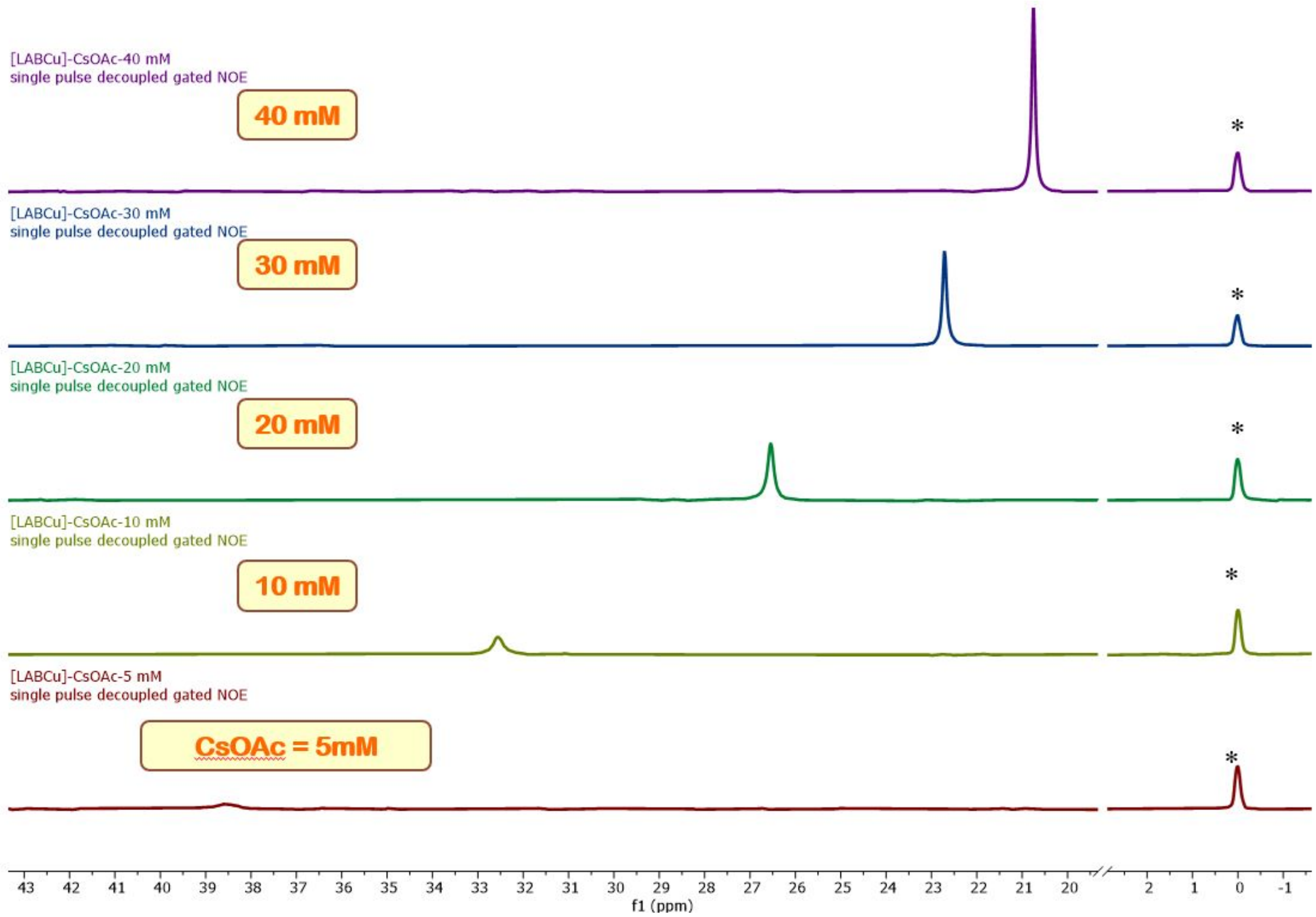

Figure S16. Illustration of [133]Cs NMR titration by treating incremental addition of CsOAc to **SP1** to form **Cs@SP1.** The resonance corresponding to pure CsOAc in MeOH, used as an internal standard, is marked with an asterisk (*)

Table S3. The experimental chemical shifts by the incremental addition of CsOAc to the tricopper Complexes **A3**, **B3** and **SP1.**

	**Chemical shifts**		
[Cs] M	**Cs@**[L_ACu]$_3$	**Cs@**[$\mathbf{L_B}$Cu]$_3$	**Cs@SP1**
0.005	44.4±0.32	40.0±0.35	38.5±0.42
0.010	36.5±0.19	33.8±0.21	32.56±0.24
0.020	30.8±0.22	27.9±0.20	26.54±0.17
0.030	27.0±0.15	24.1±0.18	22.71±0.21
0.040	24.6±0.11	21.0±0.09	20.75±0.13

DFT Computational Studies for A3B3 Coordination Driven Self-Assembly

CH_2Cl_2-Associated Dissociation

A3 + CH_2Cl_2 ⇌ **A2** + **A**(CH_2Cl_2) *(1)*

B3 + CH_2Cl_2 ⇌ **B2** + **B**(CH_2Cl_2) *(2)*

**Partial dissociation*
**Solvent-assisted accessibility*

Mixed Fragments Formation

A(CH_2Cl_2) + **B**(CH_2Cl_2) ⟶ **AB** + 2 CH_2Cl_2 *(3)*

**Thermodynamically favorable at all T*

Low Temperature Pathway

Dimeric Fragment Association

A2 + **B**(CH_2Cl_2) ⇌ **A2B** + CH_2Cl_2 *(4)*

B2 + **A**(CH_2Cl_2) ⇌ **AB2** + CH_2Cl_2 *(5)*

AB +**A**(CH_2Cl_2) ⇌ **A2B** + CH_2Cl_2 *(6)*

AB +**A**(CH_2Cl_2) ⇌ **AB2** + CH_2Cl_2 *(7)*

$\Delta H<0$, $\Delta S<0$

**Enthalpy-driven redistribution*

**Dominant at low temperature*

High Temperature Pathway

Solvent-Assisted Dissociation

A2 + 2 CH_2Cl_2 ⇌ **A2**·2CH_2Cl_2 *(8)*

B2 + 2 CH_2Cl_2 ⇌ **B2**·2CH_2Cl_2 *(9)*

$\Delta H<0$, $\Delta S<0$

A2·2CH_2Cl_2 ⇌ 2**A**(CH_2Cl_2) *(10)*

B2·2CH_2Cl_2 ⇌ 2**B**(CH_2Cl_2) *(11)*

$\Delta H>0$, $\Delta S>0$

**Entropy-driven redistribution*

**Accessible at elevated temperature*

2 **A**(CH_2Cl_2) + **B**(CH_2Cl_2) ⇌ **A2B** + 3 CH_2Cl_2 *(12)*

A(CH_2Cl_2) + 2 **B**(CH_2Cl_2) ⇌ **AB2** + 3 CH_2Cl_2 *(13)*

**Thermodynamically favorable at all T*

Scheme S2. Temperature-dependent ligand scrambling pathways in β-thioketiminato tricopper(I) clusters. Low-temperature regime (blue arrows): primarily dicopper- and monomer-mediated redistribution with enthalpy bias. High-temperature regime (red arrows): solvation-assisted monomer generation expands accessible pathways, enhancing entropy-driven scrambling. (Detailed elementary steps and structures are given in Figure S17-S19 and Table S4-S7 in the SI.)

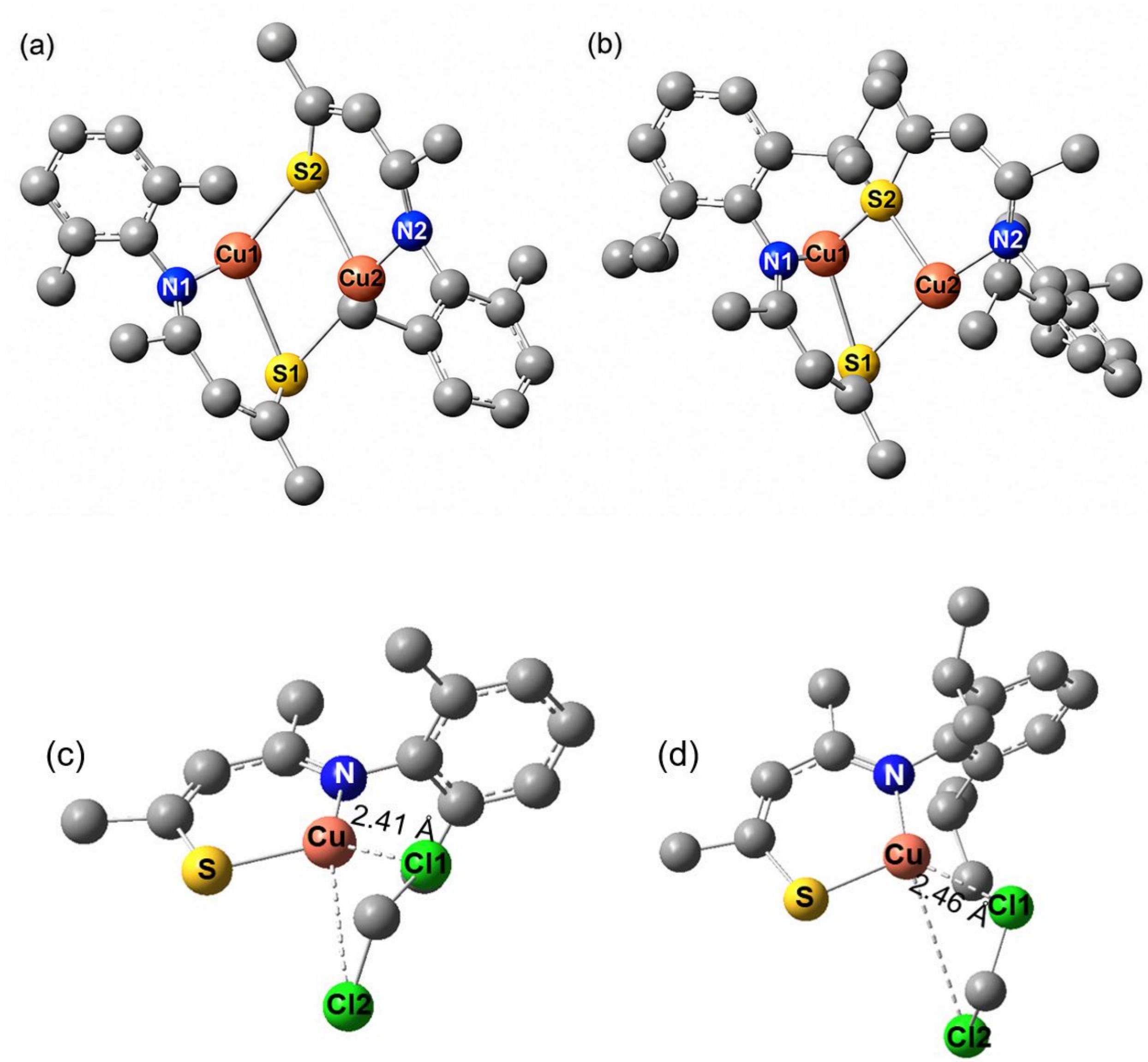

Figure S17. DFT optimized structures of the partially dissociated dicopper fragments (a) $[\mathbf{L_A}Cu]_2$ (**A2**) and (b) $[\mathbf{L_B}Cu]_2$ (**B2)**, together with the dichloromethane solvent stabilized monomeric (c) $[\mathbf{L_A}Cu\ldots CH_2Cl_2]$ (**A**(CH_2Cl_2)) and (d) $[\mathbf{L_B}Cu\ldots CH_2Cl_2]$ (**B**(CH_2Cl_2))

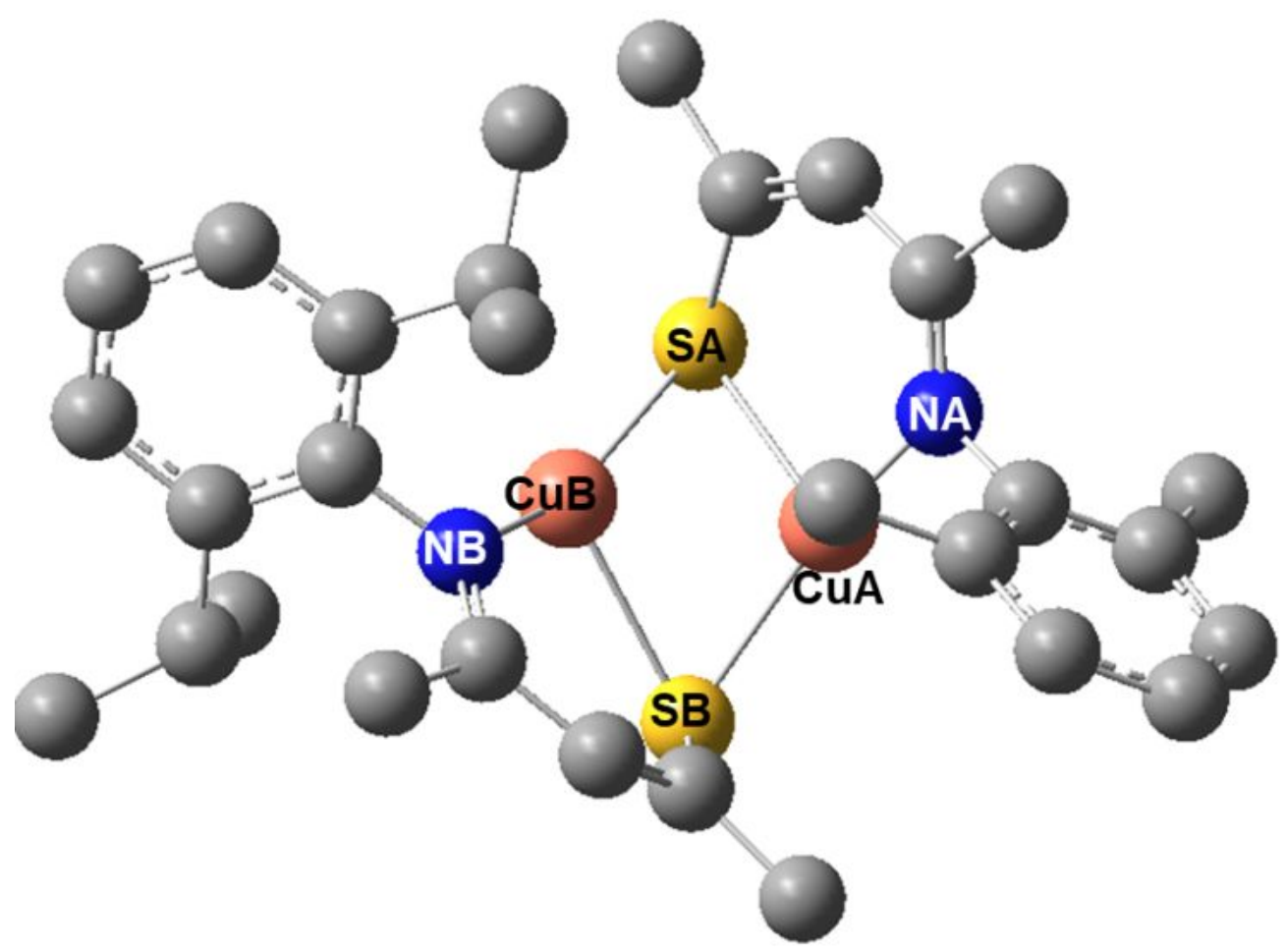

Figure S18. DFT optimized structures of the mixed dicopper fragment [$\mathbf{L_A}$CuCu$\mathbf{L_B}$] (**AB**)

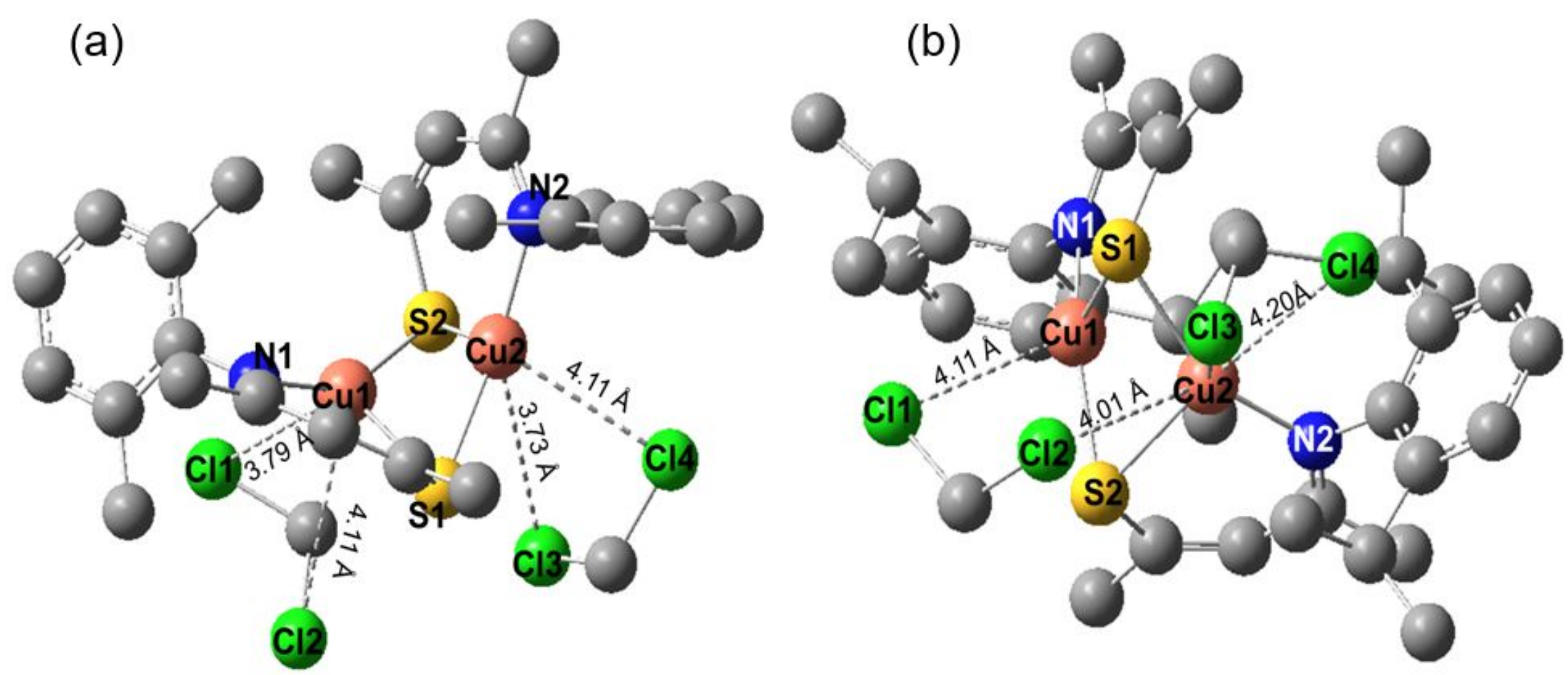

Figure S19. DFT optimized structures of the dichloromethane-solvated intermediates (a) **A2**·2CH_2Cl_2 and (b) **B2**·2CH_2Cl_2.

Table S4. DFT calculation of the thermodynamic parameters for the initial dissociation and mixed fragment formation steps

Thermodynamic parameter	**A3** + CH_2Cl_2 → **A2** + **A**(CH_2Cl_2)	**B3** + CH_2Cl_2 → **B2** + **B**(CH_2Cl_2)	**A**(CH_2Cl_2) + **B**(CH_2Cl_2) → **AB** + 2CH_2Cl_2
Sum of electronic and thermal Enthalpies ΔH (kJ/mol)	166.12	148.77	-88.21
Entropy ΔS (J/mol-K)	48.21	47.65	64.14
Thermodynamic trend	ΔH > 0; ΔS > 0	ΔH > 0; ΔS > 0	ΔH < 0; ΔS > 0

Table S5. DFT calculation of the thermodynamic parameters for the low temperature pathway

Thermodynamic Parameter	**A2**+**B**(CH_2Cl_2) →**A2B**+CH_2Cl_2	**B2**+**A**(CH_2Cl_2) →**AB2**+CH_2Cl_2	**AB**+**A**(CH_2Cl_2) →**A2B**+CH_2Cl_2	**AB**+**B**(CH_2Cl_2) →**AB2**+ CH_2Cl_2
Sum of electronic and thermal Enthalpies ΔH (kJ/mol)	-160.70	-152.33	-157.07	-152.67
Entropy ΔS (J/mol-K)	-71.68	-35.11	-51.12	-48.79
Thermodynamic trend	ΔH < 0;ΔS < 0	ΔH < 0; ΔS < 0	ΔH < 0; ΔS < 0	ΔH < 0; ΔS < 0

Table S6. DFT calculation of the thermodynamic parameters for the dimer dissociation and solvation

Thermodynamic Parameter	**A2**+2 CH_2Cl_2 → 2 **A**(CH_2Cl_2)	**B2**+2 CH_2Cl_2 → 2 **B**(CH_2Cl_2)	**A2**+2 CH_2Cl_2 → **A2**·2CH_2Cl_2	**B2**+2 CH_2Cl_2 → **B2**·2CH_2Cl_2
Sum of electronic and thermal Enthalpies ΔH (kJ/mol)	84.59	88.56	-42.84	-42.61
Entropy ΔS (J/mol-K)	-84.70	-50.46	-211.96	-179.28
Thermodynamic trend	ΔH > 0; ΔS < 0	ΔH > 0; ΔS < 0	ΔH < 0; ΔS < 0	ΔH < 0; ΔS < 0

Table S7. DFT calculation of the thermodynamic parameters for the high temperature pathway

Thermodynamic Parameter	**A2**·$2CH_2Cl_2$ →2 **A**(CH_2Cl_2)	**B2**·$2CH_2Cl_2$ → 2 **B**(CH_2Cl_2)	2 **A**(CH_2Cl_2) + **B**(CH_2Cl_2) → **A2B** + 3 CH_2Cl_2	**A**(CH_2Cl_2) + 2 **B**(CH_2Cl_2) → **AB2** + 3 CH_2Cl_2
Sum of electronic and thermal Enthalpies ΔH (kJ/mol)	127.42	131.16	-240.88	-245.28
Entropy ΔS (J/mol-K)	127.26	128.82	15.35	13.02
Thermodynamic trend	ΔH > 0; ΔS > 0	ΔH > 0; ΔS > 0	ΔH < 0; ΔS >0	ΔH < 0; ΔS < 0

DFT analysis suggests that direct CH_2Cl_2-associated dissociation of **A2** and **B2** into monomeric **A**(CH_2Cl_2) and **B**(CH_2Cl_2) fragments is thermodynamically unfavorable (ΔH > 0 and ΔS < 0). Instead, **A2** and **B2** may first undergo solvent association to form the solvated intermediates **A2**·$2CH_2Cl_2$ and **B2**·$2CH_2Cl_2$ (Figure S19 and Table S6), through processes characterized by favorable enthalpic contributions and negative entropy changes (ΔH < 0 and ΔS < 0). Subsequent dissociation of these solvated intermediates into monomeric **A**(CH_2Cl_2) and **B**(CH_2Cl_2) fragments exhibits ΔH > 0 and ΔS > 0 (Table S7), indicating that these pathways become increasingly thermodynamically accessible at elevated temperatures. The resulting increase in exchange-accessible monomeric fragments may increase the number of thermodynamically accessible redistribution states and is qualitatively consistent with the emergence of entropy-driven scrambling and near-statistical product distributions at elevated temperatures.

The experimentally observed crossover between low- and high-temperature scrambling behavior may therefore reflect a transition between distinct thermodynamic redistribution regimes. To further evaluate the entropy contributions associated with these redistribution regimes, the

overall scrambling processes at low and high temperatures were considered separately. At lower temperatures, redistribution primarily proceeds through dicopper-mediated exchange pathways, corresponding to the overall scrambling process shown in equation *(eq S1).*

$$\mathbf{A2} + \mathbf{B2} + 2\mathbf{AB} + 2\mathbf{A}(CH_2Cl_2) + 2\mathbf{B}(CH_2Cl_2) \rightleftharpoons 2\mathbf{A2B} + 2\mathbf{AB2} + 4\ CH_2Cl_2 \quad (S1)$$

Based on the configurational degeneracy associated with this redistribution process, the statistical entropy contribution estimated from S=$k_B$lnW (k_B is the Boltzmann constant) was calculated to be 36.5 J mol·K. Meanwhile, the intrinsic entropy change associated with the scrambling reaction **A3 + B3 ⇌ A2B + AB2** was computed to be −10.9 J mol·K. Accordingly, the total estimated entropy contribution at lower temperature was approximately 25.6 J mol·K, which is reasonably consistent with the value derived experimentally from VT-NMR analysis (30 J mol·K).

$$3\mathbf{A}(CH_2Cl_2) + 3\mathbf{B}(CH_2Cl_2) \rightleftharpoons 3\mathbf{A2B} + 3\mathbf{AB2} + 6\ CH_2Cl_2 \quad (S2)$$

At elevated temperatures, inclusion of the additional monomer-associated states represented by equation *(eq S2)* increases the estimated ensemble entropy contribution, consistent with the substantially larger entropy values experimentally observed in the high-temperature regime. An additional ensemble entropy contribution of 18.3 J mol·K was estimated, resulting in a total entropy contribution of approximately 43.9 J mol·K. Although this value remains substantially lower than the experimental VT-NMR result (90 ± 12 J mol·K), the discrepancy suggests that additional entropy contributions beyond the present model, such as conformational redistribution and solvent reorganization, are likely involved.

References

(1) Engel, T.; Reid, P. J. *Probability*. In *Physical Chemistry*, 3 ed.; Pearson Benjamin Cummings, 2014; pp 809-858.
